# Comparative Metabonomic Investigations of *Schistosoma japonicum* From SCID Mice and BALB/c Mice: Clues to Developmental Abnormality of Schistosome in the Immunodeficient Host

**DOI:** 10.3389/fmicb.2019.00440

**Published:** 2019-03-12

**Authors:** Rong Liu, Wen-Jun Cheng, Hong-Bin Tang, Qin-Ping Zhong, Zhen-Ping Ming, Hui-Fen Dong

**Affiliations:** ^1^School of Basic Medical Sciences, Wuhan University, Wuhan, China; ^2^Hubei Province Key Laboratory of Allergy and Immunology, School of Basic Medical Sciences, Wuhan University, Wuhan, China; ^3^Laboratory Animal Center, School of Medicine, Wuhan University, Wuhan, China

**Keywords:** *Schistosoma japonicum*, growth and development, SCID mouse, BALB/c mouse, LC-MS/MS, metabolomics

## Abstract

The growth and development of schistosome has been affected in the immunodeficient hosts. But it remains unresolved about the molecular mechanisms involved in the development and reproduction regulation of schistosomes. This study tested and compared the metabolic profiles of the male and female *Schistosoma japonicum* worms collected from SCID mice and BALB/c mice at 5 weeks post infection using liquid chromatography tandem mass spectrometry (LC-MS/MS) platform, in which the worms from SCID mice were the investigated organisms and the worms from BALB/c mice were used as the controls. There were 1015 ion features in ESI+ mode and 342 ion features in ESI- mode were identified after filtration by false discovery rate. Distinct metabolic profiles were found to clearly differentiate both male and female worms in SCID mice from those in BALB/c mice using multivariate modeling methods including the Principal Component Analysis (PCA), Partial Least Squares Discriminant Analysis (PLS-DA), and Orthogonal Partial Least Squares Discriminant Analysis (OPLS-DA). There were more differential metabolites in female worms than in male worms between SCID mice and BALB/c mice. And common and uniquely perturbed metabolites and pathways were identified among male and female worms from SCID mice when compared with BALB/c mice. The enriched metabolite sets of the differential metabolites in male worms between SCID mice and BALB/c mice included bile acid biosynthesis, taurine and hypotaurine metabolism, sphingolipid metabolism, retinol metabolism, purine metabolism, etc. And the enriched metabolite sets of differential metabolites in female worms included retinol metabolism, alpha linolenic acid and linoleic acid metabolism, purine metabolism, sphingolipid metabolism, glutamate metabolism, etc. Further detection and comparison in transcript abundance of genes of the perturbed retinol metabolism and its associated meiosis process in worms identified clues suggesting accumulated retinyl ester and perturbed meiotic process. These findings suggested an association between the schistosome with retarded growth and development in SCID mice and their perturbed metabolites and metabolic pathways, and provided a new insight into the growth and development regulation of *S. japonicum* worms from the metabolic level, which indicated great clues for discovery of drugs or vaccines against the parasites and disease with more researches.

## Introduction

Schistosomiasis, caused by infection with a parasitic blood fluke of the genus *Schistosoma*, of which *Schistosoma mansoni, S. haematobium*, and *S. japonicum* are of particular health significance, is still one of the most serious neglected tropical disease in the endemic countries (Gray et al., 2010; WHO, 2014). Unlike other trematodes, adult schistosomes are dioecious and display a fascinating codependency in that the female worm is dependent on the male worm to grow and sexually mature by residing in the male’s gynecophoral canal (Ross et al., 2002). The adult worms in pairs inhabit the mesenteric veins in the portal venous system of host, and every sexually mature female worm release thousands of eggs each day. And eggs deposited in the liver, intestinal wall and other tissues are the main pathogenic factor to the severe schistosomiasis (Ross et al., 2002). The highly evolved host-parasite relationship, especially that between schistosomes and their definitive hosts, is complex and long-lived (Halton, 1997; de Mendonca et al., 2000; Saule et al., 2002; Davies et al., 2004; Hernandez et al., 2004; You et al., 2015).

Interestingly, however, it was found that schistosomes showed retarded growth, development and reproduction in the immunodeficient mammalian hosts, resulting in attenuated pathogenesis with decreased egg-laying and hepatic granulomas formation in the hosts (Amiri et al., 1992; Davies et al., 2001; Blank et al., 2006; Cheng et al., 2008; Lamb et al., 2010; Tang et al., 2013). Some researches focusing mainly on the host revealed that the host’s factors interleukin (IL)-2 and IL-7 indirectly modulated the development of blood fluke through CD4+ T cells lymphocytes (Davies et al., 2001; Blank et al., 2006; Lamb et al., 2010). TNF was also identified to participate in maintaining the viability of adult worms with independence of the receptors TNFR1 and TNFR2 (Davies et al., 2004). However, few researches is available about the schistosomes’ molecular regulation on their growth and development.

Metabolic profile investigation is a promising approach to identify the key molecules or signaling pathways competent for addressing the phenotypic differences between worms from different hosts (Hellerman et al., 1946; Bowman et al., 1960; Thompson, 1985; Wang et al., 2004, 2008; Garcia-Perez et al., 2008; Li et al., 2009; Teng et al., 2009; Legido-Quigley, 2010; O’Sullivan et al., 2013; Sengupta et al., 2013; Zhou et al., 2015, 2016; Adebayo et al., 2018). Ultra high performance liquid chromatography and mass spectroscopy (HPLC-MS) is capable of simultaneously detecting a wide range of small molecule metabolites and providing a “metabolic fingerprint” of biological samples, and has been used as a well-established analytical tool with successful application in different fields, e.g., studying of disease progress, detection of metabolites of inborn defects, phenotypic differentiation of experimental animal models (Want et al., 2010, 2013; Cui et al., 2016). In this study, therefore, we tested and compared the metabonomic perturbations of *S. japonicum* worms with sex separation from the severe combined immunodeficient (SCID) mice at the fifth week post-infection, which were compared with those from BALB/c mice as the normal control. The results will provide new insights into understanding of the molecular regulations of growth and development of schistosomes in their hosts from the metabolic level, as well as clues for discovery of drugs and vaccines against the parasites and disease.

## Materials and Methods

### Ethics Statement

All experiments using the *S. japonicum* parasite, *Oncomelania hupensis* (*O. hupensis*) snails, and mice were performed under protocols approved by Wuhan University Center for Animal Experiments (WUCAE) according to the Regulations for the Administration of Affairs Concerning Experimental Animals of China (Ethical Approval Number: 2016025).

### Parasites and Animals

*Oncomelania hupensis* snails infected with *Schistosoma japonicum* were purchased from the Institute of Parasitic Disease Control and Prevention, Hunan Province, China. Immunocompetent BALB/c mice and severe combined immunodeficient (SCID) mice of BALB/c genetic background, approximately 6∼8 weeks old, were purchased from Beijing Hua Fu Kang Bioscience Co. Inc.^1^ via WUCAE. Cercariae were released by exposing the infected snails in aged tap water (which refers to the tap water stored in a clean plastic bucket for 3–5 days before use) under a light for a minimum of 2 h at 25°C, and were used to infected the above two kinds of mice via percutaneous exposure at approximately 40 ± 1 cercariae per mouse after 12 days of acclimatization. Intuitively, viable cercariae with intact head and tail observed under anatomical lens were counted and used for the artificial infection experiment, but the dead cercariae in motionless or cercariae with the tail off were excluded. Adult worms were collected by hepato-portal perfusion of mice with phosphate buffered solution (PBS) on the 35th day 5 weeks post infection according to our previous research (Tang et al., 2013), which had reported the detailed data illustrating the abnormal phenotype in worms from the infected SCID mice compared with those from BALB/c mice and is cited here as the research basis. The phenotypic measurement data and comparisons in their differences, therefore, were not repeatedly performed and reported here. The worms were washed with PBS twice and the male and female worms were separated manually using dissecting needle carefully under an anatomical lens (10× magnification) if necessary. Finally, the worms were collected separately with 20 worms in each aliquot, which were labeled as (1) IB-MALE for male worms collected from BALB/c mice, (2) IB-FEMALE for female worms collected from BALB/c mice, (3) IS-MALE for male worms collected from SCID mice and (4) IS-FEMALE for female worms collected from SCID mice, respectively. All the samples were frozen immediately in liquid nitrogen and then stored at -80°C until use for metabolite extraction. In addition, the blood of mice was collected and serum was isolated and stored at -80°C for serum metabolomics investigation, the paper about which was submitted elsewhere.

### Metabolite Extraction

Totally 20 frozen schistosome samples from the above four groups with five replicate samples in each group (5 replicate samples per group × 4 groups of worms = 20 samples) were sent to Wuhan Anlong Kexun Co., LTD.^2^ for metabonomic detection and analysis using HPLC-MS/MS. The LC-MS grade methanol and acetonitrile was purchased from Merck & Co., Inc., and formic acid was from Sigma-Aldrich Co. LLC. Other reagents were all analytically pure. The schistosome samples were thawed and ground after adding 0.5 ml of methanol/distilled water (8:2, v/v) with 4 μg/ml of 2-Chloro-L-phenylalanine as the internal standard substance, and were then centrifuged at 13000 rpm under 4°C for 10 min. Two hundred μl of supernatant from each sample was carefully transferred to a vial of autosampler for examination. All samples were kept at 4°C and analyzed in a random manner. Additionally, isometric supernatant from each sample of the above four groups were mixed for QC sample. The QC sample was run after every 2 tested samples to monitor the stability of the system.

### Metabolomics Analysis by HPLC-MS/MS

Liquid chromatography was performed on a 1290 Infinity UHPLC system (Agilent Technologies, Santa Clara, CA, United States). The separation of all samples was performed on an ACQUITY UPLC @HSS T3 column (Waters, United Kingdom) (100 mm ^∗^ 2.1 mm, 2.5 μm). A gradient elution program was run for chromatographic separation with mobile phase A (0.1% formic acid in water) and mobile phase B (0.1% formic acid in acetonitrile) as follows: 0∼2 min, 95%A–95%A; 2∼13 min, 95%A–5%A; 13∼15 min, 5%A–5%A. The sample injection volume was 3 μL and the flow rate was set as 0.4 mL/min. The column temperature was set at 25°C, and the post time was set as 5 min.

A 6538 UHD and Accurate-Mass Q-TOF (Agilent Technologies, Santa Clara, CA, United States) equipped with an electrospray ionization (ESI) source was used for mass spectrometric detection. The ESI mass spectra for sample analysis were acquired in both positive ion mode (ESI+) and negative ion mode (ESI-). The operating parameters were as follows: capillary, 4000 V (ESI+) or 3000 V (ESI-); sampling cone: 45 V; source temperature: 110°C (ESI+) or 120°C (ESI-); desolvation temperature: 350°C; desolvation gas, 11 L/min; source offset (skimmer1): 60 V; TOF acquisition mode: sensitivity (ESI+) or sensitivity (ESI-); acquisition method, continuum MSE; TOF mass range: 100–1000 Da; scan time: 0.2 s; collision energy function 2: trap CE ramp 20–40 eV. Quality control (QC) samples were used in order to assess the reproducibility and reliability of the LC-MS/MS system. QC samples prepared as mentioned above were used to provide a ‘mean’ profile representing all analyses encountered during the analysis. The pooled ‘QC’ samples were run before and after every two study samples to ensure system equilibration. Two reference standard compounds purine (C_5_H_4_N_4_) (with m/z 121.0509 in ESI+ mode and m/z 119.0363 in ESI- mode) and hexakis (1H,1H,3H-tetrafluoro-pentoxy)-phosphazene (C_18_H_18_O_6_N_3_P_3_F_24_) (with m/z 922.0098 in ESI+ mode and m/z 966.0007 in ESI- mode) were continuously infused into the system to allow constant mass correction during the run.

### Metabolic Data Analysis

Raw spectrometric data were uploaded to and analyzed with the MassHunter Qualitative Analysis B.04.00 software (Agilent Technologies, United States) for untargeted peak detection, peak alignment, peak grouping, normalization and integration on each full data set (study samples and QC samples). The molecular features, characterized by retention time (RT), chromatographic peak intensity, and accurate mass, were obtained by using the Molecular Feature Extractor algorithm. The features were then analyzed with the MassHunter Mass Profiler Professional software (Agilent Technologies). Only features with an intensity of ≥ 20,000 counts (approximately three times the detection limit of the LC-MS/MS instrument used in this study) that were found in at least 80% of the samples at the same sampling time point were kept for further processing. Next, a tolerance window of 0.15 min and 2 mDa was used for alignment of retention time and m/z values, and the data were also normalized by the internal standard 2-Chloro-L-phenylalanine added when sample preparation. The generalized log2 transformation and Pareto scaling (mean-centered and divided by the value range of each variable) were performed on the preprocessed data matrix prior to multivariate analysis (MVA) using Principal Component Analysis (PCA), Partial Least Squares Discriminant Analysis (PLS-DA), and Orthogonal Partial Least Squares Discriminant Analysis (OPLS-DA) to discriminate comparison groups using the function module *Statistical Analysis* on the online application *MetaboAnalyst*^3^ (Xia et al., 2015; Xia and Wishart, 2016; Chong et al., 2018). The quality of the models of PCA, PLS-DA and OPLS-DA was evaluated with the relevant parameters *R*^2^ and *Q*^2^, which were discussed elsewhere (Lee et al., 2003). And differential metabolites between groups (IS-MALE vs. IB-MALE, and IS-FEMALE vs. IB-FEMALE) were determined when variable importance in the projection (VIP) values obtained from the PLS-DA model were larger than 1.0 or the Student’s *t*-test with adjusted *P*-value (false discovery rate, FDR) of < 0.05. Fold change (FC) analysis, which was used to show how the selected differential metabolites varied between the compared groups, was also performed to further filter the features/metabolites of particular concern with an FC of ≥1.2 or ≤0.8 between the compared groups.

The structure identification of the differential metabolites was based on the methods described as follows. Briefly, the element compositions of the metabolites were first calculated with MassHunter software from Agilent based on the exact mass, the nitrogen rule, and the isotope pattern. Then, the elemental composition and exact mass were used for open source database searching, including LIPIDMAPS^4^, HMDB^5^, METLIN^6^, and MassBank^7^. Next, MS/MS experiments were performed to obtain structural information via the interpretation of the fragmentation pattern of the metabolite. The MS/MS spectra of possible metabolite candidates in the databases were also searched and matched.

*MetaboAnalyst* was used to perform metabolic pathway analysis of the differentially expressed metabolites. The identified pathways associated with the abnormal growth and development of schistosome in SCID mice are presented according to the *P*-values from the pathway enrichment analysis (*y*-axis) and pathway impact values from pathway topology analysis (*x*-axis), with the most impacted pathways colored in red color.

### Transcriptional Verification of Enzymes in Some Altered Metabolic Pathways

As a representative, genes of enzymes involved in some altered metabolic pathways with significant biological process were selected for transcriptional validation using quantitative polymerase chain reaction (qPCR). Total RNA of the schistosomes samples (IS_FEMALE, IS_MALE, IB_FEMALE, and IB_MALE), which were another replicate sample in each group collected in the same batch of experiment, was isolated using TRIzol reagent (Invitrogen, United States) according to the manufacturer’s instructions. For each sample, 1 μg of total RNA was used to synthesize the first strand cDNA using a Reverse Transcriptase Kit (TaKaRa, Dalian, China) with oligo (dT)18 primers in a final volume of 20 μl. QPCRs were performed in technological duplicate in an optical 96-well plate on *StepOne Plus* Real-Time PCR System (Applied Biosystems, Thermo Fisher Scientific, United States) using SYBR^®^ Green PCR Master Mix (TaKaRa, Dalian, China) according to the manufacturer’s instructions. Each real-time PCR reaction (in a final volume of 20 μL) contained 10 μL of 2 × SYBR^®^ Green Real-time PCR Master Mix, 0.25 μL of each primer (10 μM) (the forward and reverse primers), 1 μL of cDNA, 0.4 μL of ROX Reference dye (50×) and 8.1 μL of sterile distilled water. In parallel for each sample, 1 μL of sterile distilled water as the blank template was included as negative control. The cycling conditions included an initial denaturation and activation at 95°C for 3 min, and followed by 45 cycles at 95°C for 10 s and 60°C for 20 s. All amplifications were followed by dissociation curve analysis of the amplified products by a dissociation step (95°C for 15 s, 65°C for 10 s, 95°C for 10 s) to confirm the amplicon specificity for each gene. Specific primers of the validated genes were designed using the NCBI/Primer-BLAST^8^ with specific parameters set as PCR amplicon length of 100 – 200 bp, melting temperature (Tm) of approximately 60°C and primer pair specificity checking against Refseq mRNA (Database) of *Schistosoma* (taxid:6181) (Organism), and were commercially synthesized by Sangon Biotech (Shanghai, China) Co., Ltd. Gene expression levels were normalized to 26S proteasome non-ATPase regulatory subunit 4 (*PSMD4*)(Liu et al., 2012), and the relative expression levels were calculated using the 2ˆ(-ΔΔCt) method. Statistical significance between groups was determined using the one sample *t*-test with the cutoff *P*-value being set at 0.05.

## Results

### Metabolic Profiles

**FIGURE 1:**
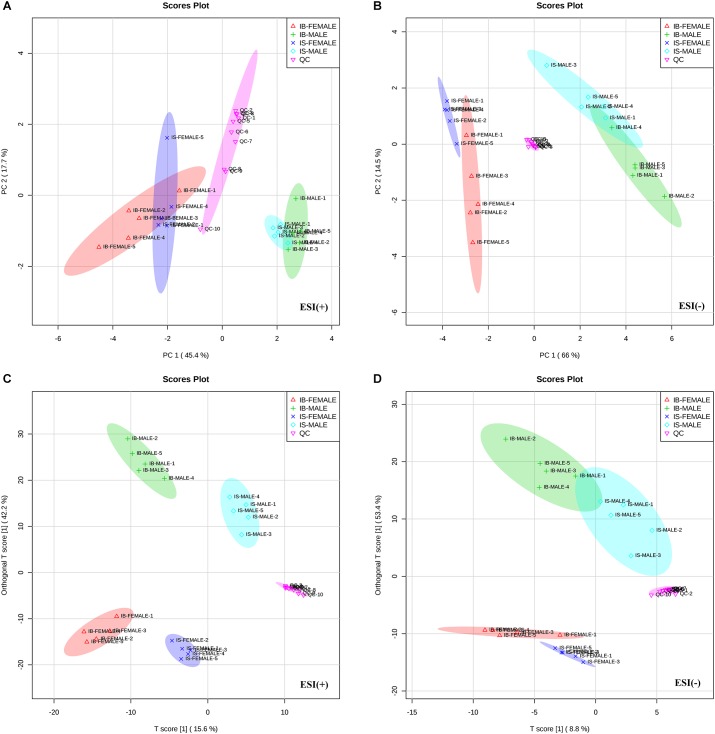
Differential metabolic profiles of male and female *S. japonicum* worms between SCID mice and BALB/c mice at 35 days post infection. Principal Component Analysis (PCA) score scatter plots of metabolites obtained from LC-MS/MS fingerprints in ESI+ **(A)** and ESI- mode **(B)**. Partial least-squares discriminant analysis (PLS-DA) separating metabolites of the four groups of worms and QC sample in ESI+ **(C)** and ESI– mode **(D)**. IB-MALE denotes the male worms from BALB/c mice, which are marked with green plus sign. IB-FEMALE denotes the female worms from BALB/c mice, which are marked with red triangles. IS-MALE denotes the male worms from SCID mice, which are marked with blue diamonds. IS-FEMALE denotes the female worms from SCID mice, which are marked with purple cross. QC denotes the quality control samples, which are marked with pink triangles. The sample size is 5 in each group of worm samples.

All total ion chromatograms (TIC) of QC samples exhibited stable retention times without obvious peaks’ drifts (Supplementary Figure S1), which indicated good capability of the LC-MS/MS based-metabolomics approach used in this study. Totally, 1015 ion features in ESI+ mode and 342 ion features in ESI- mode were obtained in all the male or female *S. japonicum* worms samples, respectively. The stability and reproducibility of the HPLC-MS/MS method was evaluated by performing PCA on all the samples, together with 10 QC samples. The QC samples are generally clustered closely to each other and are separated from the tested samples in the two-dimensional PCA score plots (Figure 1A,B) and PLS-DA score plots (Figure 1C,D), though a moderate separation among the QC samples in ESI+ mode was observed (Figure 1A), which confirms good stability and reproducibility of the chromatographic separation during the whole sequence. In addition, although the male worms (both IS-MALE and IB-MALE) were clearly separated from the female worms (both IS-FEMALE and IB-FEMALE), the male worms IS-MALE and IB-MALE were partially overlapped in the two-dimensional PCA score plots in both ion modes (Figure 1A,B), while the female worms IS-FEMALE and IB-FEMALE were completely separated in ESI- mode (Figure 1B), which indicated larger differences between male and female worms than the differences between the worms of the same sex derived from two different hosts, and larger differences between IS-FEMALE and IB-FEMALE than that between IS-MALE and IB-MALE. Similar results were also found in the two-dimensional PLS-DA models performed on all the samples, and it yielded distinct separation of the tested four groups of worms in both ESI+ (Figure 1C) and ESI- mode (Figure 1D).

### Metabolomic Profiles Distinguish Between *S. japonicum* Worms From SCID Mice and Those From BALB/c Mice

**FIGURE 2:**
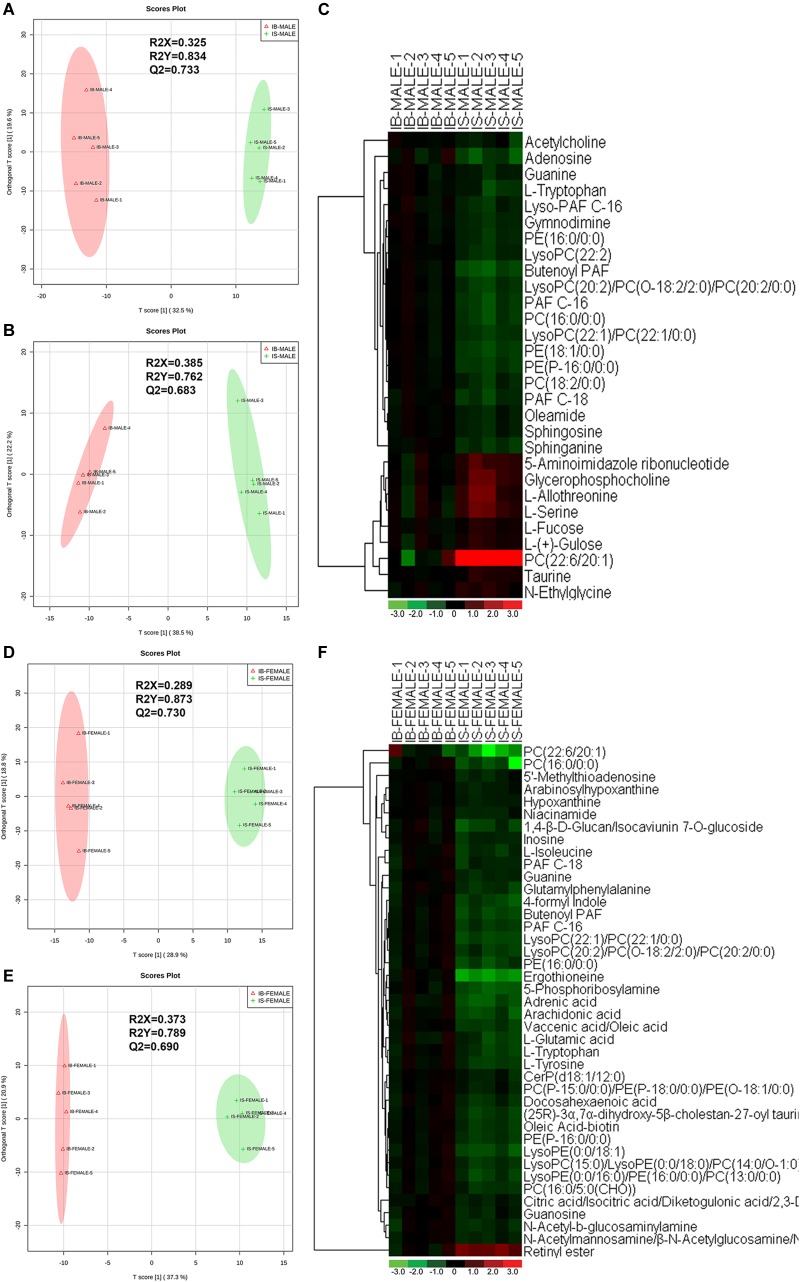
Discrimination between the *S. japonicum* worms from SCID mice and those from BALB/c mice with *S. japonicum* infection for 35 days based on ESI+ and ESI- mode-derived metabolic phenotypes and heatmaps of the differential metabolites between the compared groups. **(A,B)**: Orthogonal partial least-squares discriminant analysis (OPLS-DA) score plots in ESI+ mode **(A)** and ESI– mode **(B)** for comparison between male worms from SCID mice and those from BALB/c mice (IB-MALE denotes the male worms from BALB/c mice and IS-MALE denotes the male worms from SCID mice). **(C)**: Heatmap of the differential metabolites between male worms from SCID mice and those from BALB/c mice. **(D,E)**: Orthogonal partial least-squares discriminant analysis (OPLS-DA) score plots in ESI+ mode **(D)** and ESI– mode **(E)** for comparison between female worms from SCID mice and those from BALB/c mice (IB-FEMALE denotes the female worms from BALB/c mice and IS-FEMALE denotes the female worms from SCID mice). **(F)**: Heatmap of the differential metabolites between female worms from SCID mice and those from BALB/c mice. For the heatmaps, normalized signal intensities (log2 transformed and row adjustment) are visualized as a color spectrum and the scale from least abundant to highest ranges is from –3.0 to 3.0 as shown in the colorbar. Green indicates decreased expression, whereas red indicates increased expression of the detected metabolites between compared groups. The sample size is 5 in each group of worm samples.

Correlation analysis, PLS-DA permutation test (Supplementary Figures S2A,C) and score scatter plots for two-dimensional OPLS-DA model in both ESI+ mode (Figure 2A) and ESI- mode (Figure 2B) showed good discrimination between the male worms from SCID mice and those from BALB/c mice (IS-MALE vs. IB-MALE), which was also demonstrated in the heatmap based on the differential metabolites of the 10 male worms samples (Figure 2C). Likewise, Correlation analysis, PLS-DA permutation test (Supplementary Figures S2B,D) and the score plots for two dimensional OPLS-DA mode in both ESI+ mode (Figure 2D) and ESI- mode (Figure 2E) also showed distinct group separation between the female worms samples from SCID mice and those from BALB/c mice (IS-FEMALE vs. IB-FEMALE), which was further supported by the heatmap constructed based on the 10 female worms samples (Figure 2F).

### Patterns of Metabolites With Differential Amount in Schistosome

**FIGURE 3:**
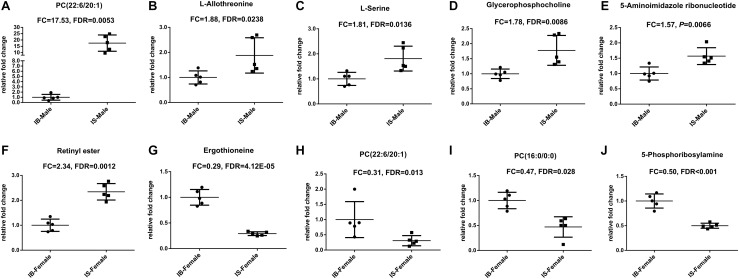
Scatter plots of differential metabolites with FC ≥ 1.5 or FC ≤ 0.5 in comparison groups. **(A–E)**: Differential metabolites between IS-MALE vs. IB-MALE; **(F–J)**: Differential metabolites between IS-FEMALE vs. IB-FEMALE. The sample size is 5 in each group of worm samples.

Twenty-nine differential ion features/metabolites (FDR < 0.05, and FC ≥ 1.2 or ≤ 0.8), with nine increased and twenty decreased, were identified between IS-MALE vs. IB-MALE (Supplementary Table S1 and Figure 2C). Five of the increased metabolites, PC (22:6/20:1) (which was traditionally named as lecithin), L-allothreonine, L-serine, glycerophosphocholine, and 5-aminoimidazole ribonucleotide, even had an FC > 1.5, particularly for PC (22:6/20:1) involved in the glycerophospholipid metabolism having an FC of 17.53 between IS-MALE vs. IB-MALE (Figure 3A–E). None of the twenty decreased metabolites had an FC < 0.5 between IS-MALE vs. IB-MALE. Meanwhile, 41 differential features/metabolites were identified between IS-FEMALE and IB-FEMALE (Supplementary Table S2 and Figure 2F). Retinyl ester, a metabolite of the retinol metabolism pathway, is the only metabolite that showed up-regulated between IS-FEMALE and IB-FEMALE. Four of the remained forty decreased metabolites, 5-phosphoribosylamine, PC(16:0/0:0), PC(22:6/20:1) and ergothioneine, even had an FC < 0.5 between IS-FEMALE and IB-FEMALE (Figure 3F–J).

**FIGURE 4:**
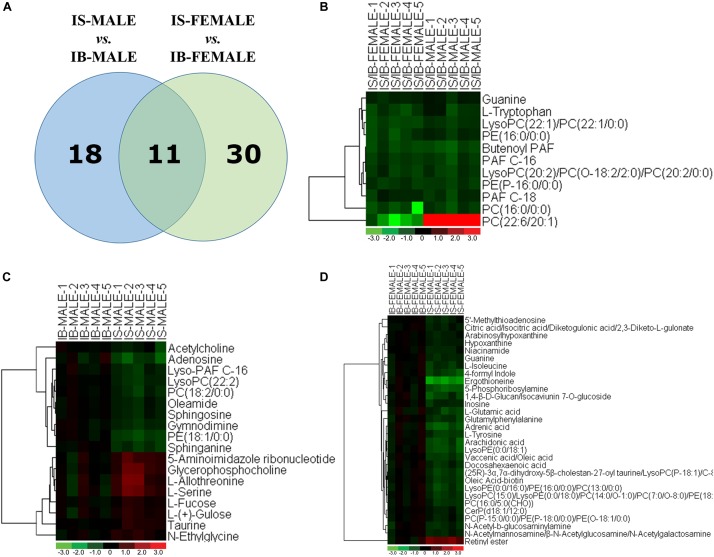
Comparison of metabolomics between the *S. japonicum* worms from SCID mice and those from BALB/c mice with *S. japonicum* infection for 35 days heatmaps of their differential metabolites. **(A)**: Differential metabolites across comparison groups showing unique and common metabolites between IS-MALE vs. IB-MALE and IS-FEMALE vs. IB-FEMALE. Venn diagram displays comparatively the differentially expressed metabolites. All the differentially expressed metabolites are clustered into two comparison groups represented by two circles. The sum of all the figures in one circle represents the number of differentially expressed metabolites in one comparison group (e.g., IS-MALE vs. IB-MALE). The overlapping part of the two circles represents the number of differentially expressed metabolites shared between the two comparison groups. The single-layer part represents the number of metabolites distinctly found in a certain comparison group. **(B–D)**: Heatmap of the common **(B)** and distinct [**(C)** is for male worms and **(D)** is for female worms] differential metabolites between male worms and female worms. For the heatmaps, normalized signal intensities (log2 transformed and row adjustment) are visualized as a color spectrum and the scale from least abundant to highest ranges is from –3.0 to 3.0 as shown in the colorbar. Green indicates decreased expression, whereas red indicates increased expression of the detected metabolites between compared groups. The sample size is 5 in each group of worm samples.

Comparison of differential metabolic profiles between IS-MALE vs. IB-MALE and IS-FEMALE vs. IB-FEMALE found that eleven features/metabolites were common in their differential metabolites (Table 1 and Figure 4A,B). Most of the common differential metabolites in both male and female worms had the similar decrease trend in the SCID mice, except that PC(22:6/20:1) increased in male worms but decreased in female worms in SCID mice compared with BALB/c mice (Table 1 and Figure 4B). After removing the common differential metabolites, eighteen differential metabolites were distinct in IS-MALE vs. IB-MALE (Table 2 and Figure 4C), and 30 differential metabolites were distinct in IS-FEMALE vs. IB-FEMALE (Table 3 and Figure 4D), which is more than male worms.

**Table 1:** List of the common differential metabolites in male and female worms from SCID mice.

ESI Mode	m/z	RT (min)	Male worms			Female worms			Metabolites
					
			*P*-value	FDR	FC(M_IS_/M_IB_)	*P*-value	FDR	FC(F_IS_/F_IB_)	
+	550.387	12.1807	3.21E-05	0.000546	0.54	0.000218	0.001573	0.57	Butenoyl PAF
+	152.0568	1.4347	0.022438	0.027246	0.79	0.003534	0.006597	0.74	Guanine
+	205.0975	3.9998	0.009361	0.013994	0.75	0.00142	0.003787	0.61	L-Tryptophan
+	548.3715	11.2785	0.000244	0.001152	0.65	0.001202	0.003416	0.64	LysoPC(20:2)/
									PC(O-18:2/2:0)/
									PC(20:2/0:0)
+	578.4181	13.7448	7.20E-05	0.000612	0.66	4.00E-05	0.000746	0.57	LysoPC(22:1)/
									PC(22:1/0:0)
+	524.3717	11.9213	0.000386	0.001458	0.62	0.000301	0.001573	0.63	PAF C-16
+	552.4029	13.4095	0.001334	0.004072	0.67	0.012797	0.01586	0.72	PAF C-18
+	496.3402	10.5067	0.000265	0.001152	0.62	0.024157	0.027552	0.47	PC(16:0/0:0)
+	860.6126	14.5507	0.00236	0.005349	17.53	0.009766	0.012718	0.31	PC(22:6/20:1)
+	454.2931	10.5736	0.000823	0.002798	0.72	0.00111	0.003416	0.60	PE(16:0/0:0)
+	460.2796	10.9475	1.99E-05	0.000546	0.63	0.000269	0.001573	0.65	PE(P-16:0/0:0)


*ESI mode: +, positive ion mode; -, negative ion mode. m/z, mass-to-charge ratio. RT, retention time. FDR, false discovery rate. FC, fold change.*

**Table 2:** List of the male worm-specific differential metabolites between SCID mice and BALB/c mice.

ESI Mode	m/z	RT(min)	*P* values	FDR	FC (M_IS_/M_IB_)	Metabolites
–	118.0509	0.6543	0.017517	0.023823	1.88	L-Allothreonine
–	104.0354	0.6497	0.008371	0.013552	1.81	L-Serine
+	258.1101	0.6723	0.004564	0.008621	1.78	Glycerophosphocholine
+	296.0658	0.6469	0.00484	0.008661	1.57	5-Aminoimidazole ribonucleotide
–	179.0556	0.654	0.049459	0.049459	1.27	L-(+)-Gulose
–	102.0549	0.6795	0.01748	0.023823	1.26	*N*-Ethylglycine
–	124.0058	0.6871	0.003978	0.007956	1.26	Taurine
–	199.0373	0.6377	0.041292	0.043873	1.21	L-Fucose
+	146.1176	3.6118	0.04623	0.047631	0.79	Acetylcholine
+	282.2795	9.3158	0.003731	0.007928	0.78	Oleamide
+	300.2902	9.3158	0.000153	0.001038	0.78	Sphingosine
+	482.3605	10.8804	0.009467	0.013994	0.73	Lyso-PAF C-16
+	508.3405	12.2338	0.001437	0.004072	0.73	Gymnodimine
+	576.4024	12.5121	0.000271	0.001152	0.72	LysoPC(22:2)
+	520.3399	10.1254	0.001677	0.004386	0.72	PC(18:2/0:0)
+	302.3053	9.016	0.000153	0.001038	0.67	Sphinganine
+	480.309	10.8995	4.90E-05	0.000556	0.61	PE(18:1/0:0)
+	268.1045	1.2762	0.005214	0.008864	0.57	Adenosine


*ESI mode: +, positive ion mode; -, negative ion mode. m/z, mass-to-charge ratio. RT, retention time. FDR, false discovery rate. FC, fold change.*

**Table 3:** List of the female worm-specific differential metabolites between SCID mice and BALB/c mice.

ESI Mode	m/z	RT(min)	*P*-values	FDR	FC (F_IS_/F_IB_)	Metabolites
–	301.2171	12.9477	0.000132	0.001233	2.34	Retinyl ester
+	269.0885	1.4201	0.003199	0.006398	0.80	Arabinosylhypoxanthine
-	464.3143	12.3082	0.025349	0.027741	0.80	PC(P-15:0/0:0)/PE(P-18:0/0:0)/PE(O-18:1/0:0)
+	137.046	1.4199	0.000225	0.001573	0.79	Hypoxanthine
-	191.0178	0.8004	0.008998	0.011998	0.79	Citric acid/Isocitric acid/Diketogulonic acid/2,3-Diketo-L-gulonate
-	596.3926	13.4124	0.010324	0.013139	0.79	CerP(18:1/12:0)
+	284.0993	1.4349	0.025797	0.027741	0.78	Guanosine
+	298.0974	4.0607	0.00461	0.007822	0.78	5′-Methylthioadenosine
+	123.0554	1.0077	0.001965	0.004584	0.77	Niacinamide
-	267.0717	1.4171	0.002114	0.004735	0.76	Inosine
-	256.0576	0.6944	0.007146	0.010815	0.76	*N*-Acetylmannosamine/*N*-Acetyl-*b*-D-galactosamine/Beta-*N*-Acetylglucosamine/*N*-Acetylgalactosamine
-	219.0968	0.6785	0.0246	0.027552	0.72	*N*-Acetyl-*b*-glucosaminylamine
-	327.2327	13.3711	0.00792	0.011671	0.71	Docosahexaenoic acid
-	592.3605	11.2786	0.004009	0.007241	0.69	PC(16:0/5:0(CHO))
+	132.1019	1.172	0.004177	0.00731	0.68	L-Isoleucine
-	480.309	11.876	0.00122	0.003416	0.67	LysoPC(15:0)/LysoPE(0:0/18:0)/PC(14:0/O-1:0)/PC(7:0/O-8:0)/PE(18:0/0:0)/PC(15:0/0:0)
-	293.1175	4.4634	0.003346	0.006462	0.67	Glutamylphenylalanine
-	557.3195	10.6175	0.000432	0.001862	0.66	Oleic Acid-biotin
+	182.0811	1.1304	0.001879	0.004574	0.65	L-Tyrosine
-	146.0449	0.6795	0.013028	0.01586	0.64	L-Glutamic acid
-	540.3297	10.6169	0.000736	0.002944	0.64	(25R)-3alpha,7alpha-dihydroxy-5beta-cholestan-27-oyl taurine/LysoPC(P-18:1)/C-8 Ceramide-1-phosphate
-	452.2769	10.5719	0.002999	0.006219	0.63	LysoPE(0:0/16:0)/PE(16:0/0:0)/PC(13:0/0:0)
-	281.2481	14.5325	7.09E-05	0.000794	0.63	Vaccenic acid/Oleic acid
-	535.1528	1.4173	0.001719	0.004375	0.59	1,4-beta-D-Glucan/Isocaviunin 7-*O*-glucoside
+	146.0602	4.0002	0.000858	0.003004	0.58	4-formyl Indole
-	303.2326	13.5603	0.000836	0.003004	0.56	Arachidonic acid
-	478.2923	10.9031	0.000403	0.001862	0.55	LysoPE(0:0/18:1)
-	331.2635	14.2904	0.000309	0.001573	0.51	Adrenic acid
-	264.005	0.7038	2.66E-05	0.000745	0.50	5-Phosphoribosylamine
+	230.0967	0.7226	7.35E-07	4.12E-05	0.29	Ergothioneine


*ESI mode: +, positive ion mode; -, negative ion mode. m/z, mass-to-charge ratio. RT, retention time. FDR, false discovery rate. FC, fold change.*

**FIGURE 5:**
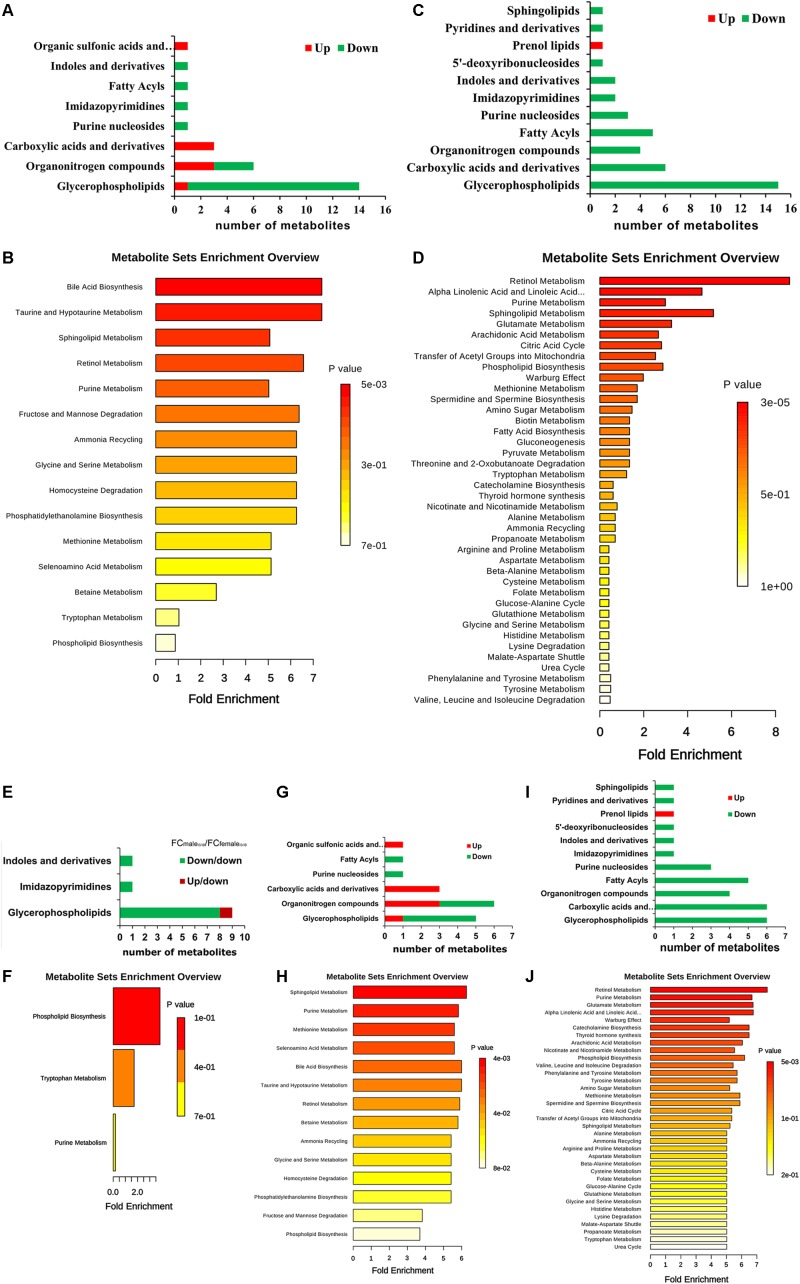
Enrichment analysis of the differential metabolites across comparison groups. **(A)**: Top 8 enriched metabolite terms of the differentially expressed metabolites of IS-MALE vs. IB-MALE. The bars on *x*-axis represent the number of metabolites for the chemical classes mentioned on the *y*-axis. **(B)**: Enriched metabolite sets of the differentially expressed metabolites of IS-MALE vs. IB-MALE. **(C)**: Top 11 enriched metabolite terms of the differentially expressed metabolites of IS-FEMALE vs. IB-FEMALE. **(D)**: Enriched metabolite sets of the differentially expressed metabolites of IS-FEMALE vs. IB-FEMALE. **(E)**: Enriched metabolite terms of the common differentially expressed metabolites between IS-MALE vs. IB-MALE and IS-FEMALE vs. IB-FEMALE. **(F)**: Enriched metabolite sets of the differentially expressed metabolites between IS-MALE vs. IB-MALE and IS-FEMALE vs. IB-FEMALE. **(G)**: Enriched metabolite terms of the differentially expressed metabolites distinct in IS-MALE vs. IB-MALE. **(H)**: Enriched metabolite sets of the differentially expressed metabolites distinct in IS-MALE vs. IB-MALE. **(I)**: Enriched metabolite terms of the differentially expressed metabolites distinct in IS-FEMALE vs. IB-FEMALE. **(J)**: Enriched metabolite sets of the differentially expressed metabolites distinct in IS-FEMALE vs. IB-FEMALE.

By searching against “The Human Metabolome Database” (HMDB^9^) for metabolite classification, “glycerophospholipids,” “organonitrogen compounds” and “carboxylic acids and derivatives” were found as the top three categories (≥3 differential metabolites involved) of differential metabolites between IS-MALE vs. IB-MALE (Figure 5A). Metabolite set enrichment analysis (MSEA^10^) found that “bile acid biosynthesis,” “taurine and hypotaurine metabolism,” “sphingolipid metabolism,” “retinol metabolism,” “purine metabolism,” “fructose and mannose degradation,” “ammonia recycling,” “glycine and serine metabolism,” “homocysteine degradation,” “phosphatidylethanolamine biosynthesis,” “methionine metabolism” and “selenoamino acid metabolism” were the prominently enriched metabolite sets (with adjusted *P*-values < 0.05) based on the differential metabolites between IS-MALE vs. IB-MALE (Supplementary Table S3 and Figure 5B). Meanwhile, “glycerophospholipids,” “carboxylic acids and derivatives,” “organonitrogen compounds,” “fatty acyls” and “purine nucleosides” were the top five enriched categories of differential metabolites between IS-FEMALE vs. IB-FEMALE (Figure 5C). And MSEA based on the differential metabolites between IS-FEMALE vs. IB-FEMALE found that “retinol metabolism,” “alpha linolenic acid and linoleic acid metabolism,” “purine metabolism,” “sphingolipid metabolism” and “glutamate metabolism” were the prominently enriched metabolite sets with raw *P*-values < 0.05 but only “retinol metabolism” has an adjusted *P*-value < 0.05 (Supplementary Table S4 and Figure 5D). Moreover, most (9/11) of the common differential metabolites between IS-MALE vs. IB-MALE and IS-FEMALE vs. IB-FEMALE belong to glycerophospholipids (Figure 5E), which was enriched by MSEA to phospholipid biosynthesis (Supplementary Table S5 and Figure 5F). The differential metabolites distinct in IS-MALE vs. IB-MALE were classified prominently into “organonitrogen compounds,” “glycerophospholipids” and “carboxylic acids and derivatives” (Figure 5G), which were enriched prominently to “sphingolipid metabolism,” “purine Metabolism,” “methionine metabolism,” “selenoamino acid metabolism,” “bile acid biosynthesis,” “taurine and hypotaurine metabolism,” “retinol metabolism” and “betaine metabolism” (Supplementary Table S6 and Figure 5H). The differential metabolites distinct in IS-FEMALE vs. IB-FEMALE were classified prominently into “glycerophospholipids,” “carboxylic acids and derivatives,” “fatty acyls,” “organonitrogen compounds” and “purine nucleosides” (Figure 5I), which were enriched prominently to “retinol metabolism,” “purine metabolism,” “glutamate metabolism,” “alpha linolenic acid and linoleic acid metabolism” and “warburg effect” (Supplementary Table S7 and Figure 5J).

### Altered Metabolic Pathways With Biological Significance

The pathway analysis performed using *MetaboAnalyst* for the involved biological pathways and biological roles of the above differentially expressed metabolites determined that the perturbed metabolic pathways reporting lower *p*-values and higher pathway impact in male worms from SCID mice compared with those from BALB/c mice mainly included arachidonic acid metabolism, alpha-linolenic acid metabolism, taurine and hypotaurine metabolism, sphingolipid metabolism, glycerophospholipid metabolism, and etc (Supplementary Table S8 and Supplementary Figure S3A). Meanwhile, more affected metabolic pathways in female worms from SCID mice compared with those from BALB/c mice were determined, and the top 5 metabolic pathways included biotin metabolism, tryptophan metabolism, purine metabolism, glyoxylate and dicarboxylate metabolism, tyrosine metabolism (Supplementary Table S9 and Supplementary Figure S3B).

Metabolic pathways analysis based on the common differential metabolites between IS-MALE vs. IB-MALE and IS-FEMALE vs. IB-FEMALE found that tryptophan metabolism, aminoacyl-tRNA biosynthesis, purine metabolism, glycerophospholipid metabolism, arachidonic acid metabolism and alpha-linolenic acid metabolism were commonly perturbed in both male and female worms from SCID mice compared with BALB/c mice (Supplementary Table S10 and Supplementary Figure S3C). Metabolic pathways analysis based on the differential metabolites distinct in IS-MALE vs. IB-MALE found their involved metabolic pathways included sphingolipid metabolism, glycerophospholipid metabolism, taurine and hypotaurine metabolism, purine metabolism, glycine/serine/threonine metabolism, cysteine and methionine metabolism, cyanoamino acid metabolism, glyoxylate and dicarboxylate metabolism and aminoacyl-tRNA biosynthesis (Supplementary Table S11 and Supplementary Figure S3D). More metabolic pathways based on the differential metabolites distinct in IS-FEMALE vs. IB-FEMALE were found and the top five metabolic pathways were arachidonic acid metabolism, glycerophospholipid metabolism, glycosylphosphatidylinositol (GPI)-anchor biosynthesis, alpha-linolenic acid metabolism and glyoxylate and dicarboxylate metabolism (Supplementary Table S12 and Supplementary Figure S3E).

### QPCR Examination of Enzymes in Retinol Metabolism and Meiosis

**FIGURE 6:**
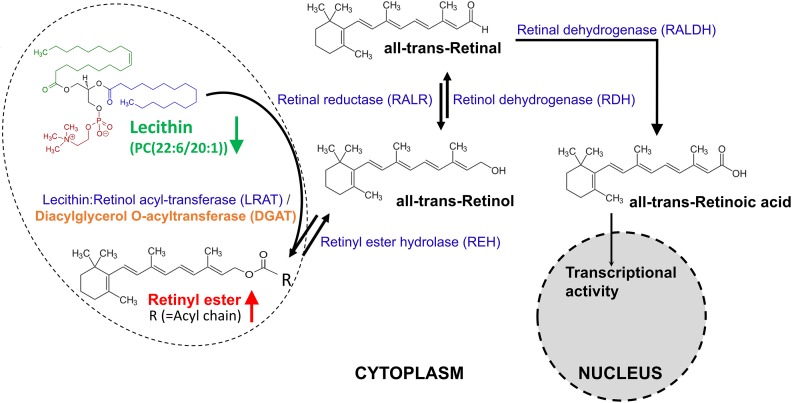
The metabolism of retinoids. Retinal can be originally formed by the cleavage of proretinoid carotenoids such as beta-carotene by the enzyme BCMO1 (not shown here). Retinol is formed by the reversible reduction of retinal by one of the retinal reductase family members. The enzyme lecithin:retinol acyl-transferase (LRAT) [with an ortholog gene called diacylglycerol *O*-acyltransferase (DGAT) in *Schistosoma*] synthesizes retinyl esters by transferring a fatty acyl moiety from the sn-1 position of membrane phosphatidyl choline such as lecithin [PC(22:6/20:1)] to retinol. Unesterified retinol is liberated from retinyl ester stores by the catalysis of a retinyl ester hydrolase (REH). Retinol is oxidized by catalysis of one retinol dehydrogenase (RDH) to retinal, which is then irreversibly oxidized by one retinal dehydrogenase (RALDH) to form transcriptionally active retinoic acid. Retinoic acid is finally oxidized/catabolized to more water-soluble hydroxy- and oxo- forms by one of several cytochrome P450 enzyme family members.

**FIGURE 7:**
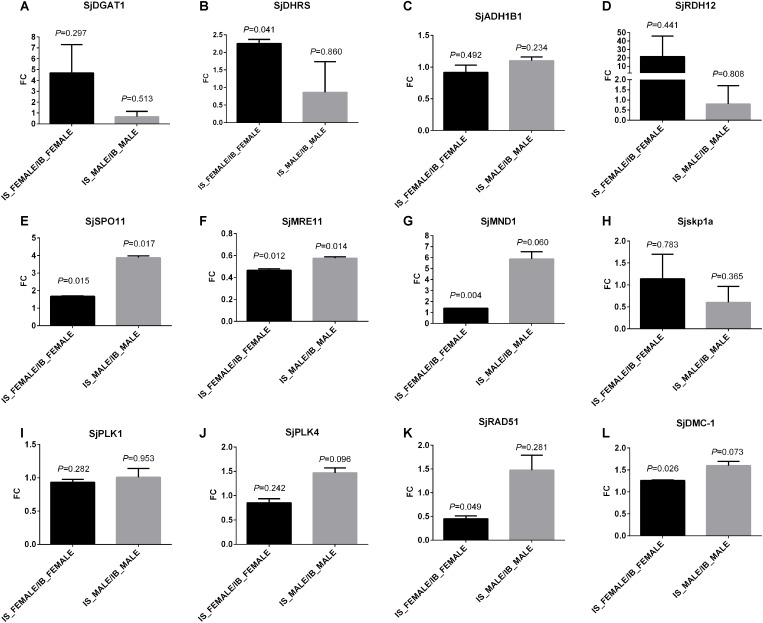
Transcriptional verification of some genes involved in retinol metabolism and the associated meiosis process by qPCR. The tested four genes in “retinol metabolism” are: **(A)** *S. japonicum* diacylglycerol *O*-acyltransferase 1 (*SjDGAT1*), **(B)** short chain dehydrogenase/reductase (*SjDHRS*), **(C)** aldehyde dehydrogenase 1B1 precursor (*SjALDH1B1*), and **(D)** retinol dehydrogenase 12 (*SjRDH12*). The tested eight genes involved in meiosis are: **(E)** *S. japonicum* meiotic recombination protein SPO11 (*SjSPO11*), **(F)** double-strand break repair protein MRE11A *(SjMRE11*), **(G)** meiotic nuclear division protein 1-like protein (*SjMND1*), **(H)** S-phase kinase-associated protein 1A (*Sjskp1a*), **(I)** polo-like kinase 1 (*SjPLK1*), **(J)** polo-like kinase 4 (*SjPLK4*), **(K)** DNA repair protein RAD51 (*SjRAD51*), and **(L)** meiotic recombinase DMC1 (*SjDMC1*). S_F: IS-FEMALE, B_F: IB-FEMALE, S_M: IS-MALE, and B_M: IB-MALE. Two duplicate examinations were performed for each group of worm samples.

To find evidences in transcript level, four genes in “retinol metabolism” (Figure 6) and eight genes in the associated meiosis process with “retinol metabolism” (O’Byrne and Blaner, 2013), as representatives, were selected for transcript abundance comparison. They are *S. japonicum* diacylglycerol *O*-acyltransferase 1 (*SjDGAT1*), retinol dehydrogenase 12 (*SjRDH12*), short chain dehydrogenase/reductase (*SjDHRS*), aldehyde dehydrogenase 1B1 precursor (*SjALDH1B1*) involved in retinol metabolism, and meiotic recombination protein SPO11 (*SjSPO11*), double-strand break repair protein MRE11A *(SjMRE11*), S-phase kinase-associated protein 1A (*Sjskp1a*), meiotic nuclear division protein 1-like protein (*SjMND1*), polo-like kinase 1 (*SjPLK1*), polo-like kinase 4 (*SjPLK4*), DNA repair protein RAD51 (*SjRAD51*), and meiotic recombinase DMC1 (*SjDMC1*). These genes and their full or partial mRNA sequences were obtained by name searching or sequence blast from a known gene sequence of other species against the public databases NCBI and WormBase ParaSite. The detailed information of primers of the tested genes are listed in Supplementary Table S13. The qPCR results found the transcript levels of *SjDGAT1, SjDHRS* and *SjRDH12* in “retinol metabolism” were elevated in female worms from SCID mice when compared with those in female worms from BALB/c mice (Figure 7A,B,D), though only *P*-value of *SjDHRS* was smaller than 0.05, but not in male worms from SCID mice. No significant difference was detected in the level of *SjADH1B1* in both female and male worms from SCID mice when compared with those from BALB/c mice (Figure 7C). The transcript levels of *SjSPO11, SjMND1* and *SjDMC1* were elevated in female worms from SCID mice when compared with that in female worms from BALB/c mice (Figure 7E,G,L), *SjMRE11* decreased almost half in female worms from SCID mice when compared with that in female worms from BALB/c mice (Figure 7F). Meanwhile, *SjSPO11, SjMND1, SjPLK4, SjRAD51*, and *SjDMC1* were elevated in male worms from SCID mice (Figure 7E,G,J–L), while *SjMRE11* and *Sjskp1a* decreased in male worms from SCID mice (Figure 7F,H) though some *P*-values were larger than 0.05. No significant difference was detected in the level of *SjPLK1* in both female and male worms from SCID mice when compared with those from BALB/c mice (Figure 7I).

## Discussion

In this study, an untargeted LC-MS/MS-based high-resolution metabolomic investigation was performed and distinct bio-signatures in the metabolic profiles of male and female *S. japonicum* worms in SCID mice were found when compared with those in BALB/c mice, respectively. In the results, MVA by both PCA and PLS-DA found larger differences between IS-FEMALE and IB-FEMALE than that between IS-MALE and IB-MALE. This indicates the female schistosome worms were affected more severely than the male worms in SCID mice, which was verified by the subsequent finding that more differential metabolites were acquired in IS-FEMALE vs. IB-FEMALE than IS-MALE vs. IB-MALE. This is expectable and reasonable as the growth and development of female worms were affected by male worms as well as the host’s factors, i.e., the sexual maturation of female worms depends on pairing with male worms (Shaw et al., 1977; Popiel, 1986; Gupta and Basch, 1987; Boissier and Mone, 2001; Kunz, 2001; Osman et al., 2006; Quack et al., 2006; LoVerde et al., 2009; Wang et al., 2017). These differential metabolites common and distinct in male worms or female worms in SCID mice compared with BALB/c mice may be associated with the abnormal growth and development of worms in SCID mice, and the differential metabolites distinct in male worms or female worms should be associated with larger differences between IS-FEMALE and IB-FEMALE than those between IS-MALE and IB-MALE.

In the list of differential metabolites of IS-FEMALE vs. IB-FEMALE, retinyl ester was the only up-regulated metabolite of female worms from SCID mice, which was enriched in “retinol metabolism.” Numerous researches reported that the retinol metabolism, in which retinyl ester is involved, regulates gametogenesis and reproduction by the product transcriptionally active retinoic acid (Chung and Wolgemuth, 2004; Koubova et al., 2006; Alsop et al., 2008; Kim et al., 2008; Amory et al., 2011). In the retinol metabolism pathway of animals, all-trans retinyl esters in the body is formed by transferring a fatty acyl moiety from the sn-1 position of membrane phosphatidyl choline [e.g., PC(22:6/20:1), traditionally named as lecithin] to all-trans-retinol under the catalysis of lecithin:retinol acyltransferase (LRAT), whose ortholog in *Schistosoma* is diacylglycerol *O*-acyltransferase 1 (DGAT1). Unesterified all-trans-retinol, which could be reversibly liberated from all-trans retinyl esters stores through the action of a retinyl ester hydrolase (REH), is oxidized by one retinol dehydrogenase (RDH) to all-*trans*-retinal, which can be also reversibly transformed to all-trans-retinol by the catalysis of retinal reductase (RALR). All-trans-retinal, which is originally derived from the decomposition of proretinoid carotenoids such as dietary β-carotene, is then irreversibly oxidized by one retinal dehydrogenase (RALDH) to form transcriptionally active all-trans retinoic acid (O’Byrne and Blaner, 2013). LRAT is a key enzyme involved in retinoids homeostasis and is regulated in response to retinoic acid, and it can also negatively regulate retinoic acid biosynthesis by diverting retinol away from oxidative activation (O’Byrne and Blaner, 2013). Therefore, we speculate higher level of retinyl ester found in the female worms from SCID mice logically means lower level of lecithin, which should be consumed to synthesize retinyl ester. What was consistent with this inference in the results was that PC(22:6/20:1) (HMDB0008735, with full name as 1-docosahexaenoyl-2-eicosenoyl-sn-glycero-3-phosphocholine, or traditional name as lecithin) happened to be decreased in the female worms from SCID mice when compared with those from BALB/c mice (Figure 6). So, we speculated that the accumulated retinyl ester in the female worms from SCID mice resulted in insufficient formation of the active retinoic acid. It is known that retinoic acid is the meiosis-inducing factor in both sexes, and inhibition of retinoic acid biosynthesis would markedly suppresses gametogenesis (Kim and Griswold, 1990; Chung and Wolgemuth, 2004; Bowles et al., 2006; Koubova et al., 2006; Doyle et al., 2007; Alsop et al., 2008; Anderson et al., 2008; Kim et al., 2008; Amory et al., 2011; Duester, 2013; Nicholls et al., 2013; Paik et al., 2014; Liu R. et al., 2018; Nourashrafeddin and Hosseini Rashidi, 2018). In addition, overexpression of LRAT will favor retinyl ester formation, which would disrupt retinol homeostasis and interrupt the ability of downstream metabolites to regulate transcription of genes involved in various biological processes. Various cancer cells have been found to have low levels of LRAT and retinyl ester levels. Overexpression of LRAT or increased level of retinyl esters themselves makes cells more sensitive to carcinogen-induced tumorigenesis and leison (Tang et al., 2009; Ghosh, 2014). So, insufficient retinoic acid biosynthesis due to prevailing retinyl ester formation could be a significant cause for the retarded development and declined fertility appeared in female worms from SCID mice in this study. QPCR verification in transcript abundance of some genes in “retinol metabolism” and the associated meiosis process found *SjDGAT1*, which encodes a protein catalyzing the conversion of all-*trans*-retinol and lecithin to retinyl ester, was elevated in female worms from SCID mice when compared with those in female worms from BALB/c mice. This was just consistent with the metabolic finding that there was higher level of retinyl ester in female worms from SCID mice. In addition, *SjSPO11*, which encodes a protein involved in the creation of double stranded breaks in the DNA in the early stages of meiotic recombination, was found with higher level in female worms as well as male worms from SCID mice when compared with those from BALB/c mice. Meanwhile, *SjMRE11* and *SjRAD51*, which encode enzymes involved in DNA double-strand break repair, were found with lower level in female worms from SCID mice when compared with those from BALB/c mice. So, both of these were consistent with the above speculation that prevailing retinyl ester formation could lead to retarded development and declined fertility in worms due to affected meiotic process.

Meanwhile, lecithin is a source of several active compounds: choline and its metabolites are needed for several physiological purposes, including cell membrane signaling and cholinergic neurotransmission during growth and reproduction (Attia et al., 2009; Attia and Kamel, 2012). So, excessive lecithin consumption in retinyl ester formation probably lead to worse effect in the development and reproduction of schistosomes besides insufficient retinoic acid biosynthesis. In contrast, however, an extremely higher level of lecithin was detected in the male worms from SCID mice than BALB/c mice, with a relative fold as high as 17.53 for IS-MALE vs. IB-MALE. It is known that the schistosomes are dioecious trematodes, and embracing with the male worm by residing in the male’s gynaecophoric channel is crucial for the female worm to grow and sexually mature (Gryseels et al., 2006). So, we speculated that insufficient interaction between the male and female worms, which manifested as decreased percent of worm pairs reported in our previous research (Tang et al., 2013), resulted in insufficient material exchange or transfer between them, such as the probable lecithin transfer from male worms to female worms. Similar alterations in fatty acyls, glycerophospholipids, purine nucleosides, imidazopyrimidines and indoles and derivatives were detected in both male and female worms from SCID mice when compared with BALB/c mice, but opposite alterations were detected in carboxylic acids and derivatives (Figure 5A,C,E). Common decrease in glycerophospholipids synthesis with lysoPCs, lysoPEs and PCs as the top three alter metabolites, one of the main functions of which is to serve as a structural component of biological membranes (Farooqui et al., 2000; Zufferey et al., 2017), indicated attenuated parasite establishment with smaller body size and attenuated reproduction due to potentially deficient glycerophospholipids in worms from SCID mice.

Sphingolipids are commonly believed to protect the cell surface against harmful environmental factors (Hannun and Obeid, 2008; Bartke and Hannun, 2009), and arachidonic acid is involved in cellular signaling as a lipid second messenger as a polyunsaturated fatty acid present in the phospholipids (Wang et al., 2006; Fukaya et al., 2007). Their involved metabolic pathways of important biological significance such as sphingolipid metabolism and arachidonic acid metabolism were also found abnormal with decreased sphingolipids in male and arachidonic acid in female worms from SCID mice, respectively, which may be also associated with the developmentally stunted worms.

Furthermore, the level of tryptophan, an essential amino acid, was found decreased in both male and female worms from SCID mice. Tryptophan acts as a precursor for the synthesis of the neurotransmitters melatonin and serotonin and then, any reduction in tryptophan will lead to a number of conditions or diseases, e.g., dermatitis and psychiatric symptom – depression in animals, it seems in this study to contribute to inhibiting of the growth and reproduction of worms finally (Jing et al., 2009; Shen et al., 2012; Tsuji et al., 2013; Liu et al., 2015, 2017; Zhang et al., 2018). Ergothioneine is a product of plant origin that accumulates in animal tissues and a naturally occurring metabolite of histidine that has antioxidant properties though its physiological role *in vivo* is undetermined (Aruoma et al., 1999). Decrease of ergothioneine was found in female worms from SCID mice, which indicated increased susceptibility to oxidative damage in them (Zhu et al., 2011; Cheah and Halliwell, 2012; Cheah et al., 2017).

## Conclusion

The identified differential metabolites and their involved metabolic pathways are likely associated with the abnormalities in growth and development of *S. japonicum* worms in SCID mice when compared with those in BALB/c mice. Differential alterations in metabolic profiles between male and female worms from SCID mice when compared with BALB/c mice indicated the degree and mechanism of the influence the host on male and female worms were different. Our data has demonstrated the great ability of LC-MS/MS-based metabolomics to detect a broad range of differential metabolites in worms that strongly distinguished between their different hosts – the SCID mice and BALB/c mice. The above mentioned differential metabolites, together with the others not mentioned here in particular, need further verification and investigations for their underlying mechanisms in the regulation of growth and development of schistosomes. As a result, this study, which described the application of metabolomics method to better understand *S. japonicum* biology, will greatly facilitate the discovery of new drugs and vaccines against schistosomes and schistosomiasis.

## Data Availability

All datasets generated for this study are included in the manuscript and/or the Supplementary Files.

## Author Contributions

RL conceived and designed the experiments. RL, W-JC, H-BT, and Q-PZ performed the experiments. RL, Z-PM, and H-FD contributed reagents, materials, and analysis tools. RL analyzed the data and wrote the manuscript. RL and H-FD critically revised the manuscript. All authors read and approved the final version of the manuscript.

## Conflict of Interest Statement

The authors declare that the research was conducted in the absence of any commercial or financial relationships that could be construed as a potential conflict of interest.

## Footnotes

1 http://www.hfkbio.com/

2 www.anachro.com.cn

3 http://www.metaboanalyst.ca/

4 http://www.lipidmaps.org/

5 http://www.hmdb.ca/

6 http://metlin.scripps.edu/

7 http://www.massbank.jp/

8 https://www.ncbi.nlm.nih.gov/tools/primer-blast/index.cgi?LINK_LOC=BlastHome

9 http://www.hmdb.ca/

10 http://www.metaboanalyst.ca/

## Supplementary Material

FIGURE S1 The stacked total ion chromatograms of QC sample. **(A)** The stacked total ion chromatogram of QC sample in ESI+ mode. **(B)** The stacked total ion chromatogram of QC sample in ESI- mode. The bars on *x*-axis represent the retention time (0∼15 min) and the bars on *y*-axis represent the total ion strength. The sample size is 5 in each group of worm samples.

FIGURE S2 Correlation analysis and PLS-DA permutation test between samples. **(A)** Correlation Analysis between male worm samples. **(B)** Correlation Analysis between female worm samples. **(C)** PLS-DA Permutation test of male worm samples. **(D)** PLS-DA Permutation test of female worm samples. Distance measurement for Correlation Analysis is performed by Spearman rank correlation. Permutation numbers for Permutation test equal to 1000.

FIGURE S3 Summary of the aberrant metabolic pathways based on the significantly altered metabolites of the comparison groups as analyzed by Pathway Analysis of *MetaboAnalyst*. Plots show the matched pathways depicted according to *P*-value from pathway enrichment analysis and pathway impact score from pathway topology analysis. **(A)** Enriched metabolic pathways based on the differential metabolites between IS-MALE vs. IB-MALE. **(B)** Enriched metabolic pathways based on the differential serum metabolites between IS-FEMALE vs. IB-FEMALE. **(C)** Enriched metabolic pathways based on the differential metabolites common in IS-MALE vs. IB-MALE and IS-FEMALE vs. IB-FEMALE. **(D)** Enriched metabolic pathways based on the differential metabolites distinct in IS-MALE vs. IB-MALE. **(E)** Enriched metabolic pathways based on the differential metabolites distinct in IS-FEMALE vs. IB-FEMALE. Color gradient and circle size indicate the significance of the pathway ranked by *P*-value (yellow: higher *P*-values and red: lower *P*-values) and pathway impact score (the larger the circle the higher the impact score), respectively. Significantly affected pathways with low *P*-value and high pathway impact score are identified by name.

TABLE S1 Differential metabolites of male worms from SCID mice compared with those from BALB/c mice. This table contains a list of the differential metabolites between male worms from SCID mice and male worms from BALB/c mice.

TABLE S2 Differential metabolites of female worms from SCID mice compared with those from BALB/c mice. This table contains a list of the differential metabolites between female worms from SCID mice and female worms from BALB/c mice.

TABLE S3 Metabolite set enrichment of the differential metabolites of male worms between SCID mice and BALB/c mice. Metabolite sets are enriched of the differential metabolites between male worms from SCID mice and male worms from BALB/c mice.

TABLE S4 Metabolite set enrichment of the differential metabolites of female worms between SCID mice and BALB/c mice. Metabolite sets are enriched of the differential metabolites between female worms from SCID mice and female worms from BALB/c mice.

TABLE S5 Metabolite set enrichment of the common differential metabolites in male and female worms from SCID mice. Metabolite sets are enriched of the common differential metabolites between male worms and female worms from SCID mice compared with those from BALB/c mice.

TABLE S6 Metabolite set enrichment of the male worm-specific differential metabolites between SCID mice and BALB/c mice. Metabolite sets are enriched of the male worm-specific differential metabolites between SCID mice and BALB/c mice.

TABLE S7 Metabolite set enrichment of the female worm-specific differential metabolites of those between SCID mice and BALB/c mice. Metabolite sets are enriched of the female worm-specific differential metabolites between SCID mice and BALB/c mice.

TABLE S8 Pathway analysis of the differential metabolites of male worms between SCID mice and BALB/c mice. This list contains the enriched metabolic pathways of the differential metabolites of male worms between SCID mice and BALB/c mice.

TABLE S9 Pathway analysis of the differential metabolites of female worms between SCID mice and BALB/c mice. This list contains the enriched metabolic pathways of the differential metabolites of female worms between SCID mice and BALB/c mice.

TABLE S10 Pathway analysis of the common differential metabolites in male and female worms from SCID mice. This list contains the enriched metabolic pathways of the common differential metabolites between male worms and female worms from SCID mice compared with those from BALB/c mice.

TABLE S11 Pathway analysis of the male worm-specific differential metabolites between SCID mice and BALB/c mice. This list contains the enriched metabolic pathways of the male worm-specific differential metabolites between SCID mice and BALB/c mice.

TABLE S12 Pathway analysis of the female worm-specific differential metabolites between SCID mice and BALB/c mice. This list contains the enriched metabolic pathways of the female worm-specific differential metabolites between SCID mice and BALB/c mice.

TABLE S13 Information of qPCR primers of the tested genes 1 involved in retinol metabolism and meiosis. This list contains the detailed information of qPCR primers of the tested genes involved in retinol metabolism and its regulated process of great biological significance - meiosis.
