# Supplementary figures and images for: Comparative Metabonomic Investigations of Schistosoma japonicum From SCID Mice and BALB/c Mice: Clues to Developmental Abnormality of Schistosome in the Immunodeficient Host
Source: Front Microbiol. 2019 Mar 12;10:440. doi: 10.3389/fmicb.2019.00440 (PMC6423161; doi:10.3389/fmicb.2019.00440)

QC-pos-10\_1\_ESI+\_TIC

**A**

Figure S1

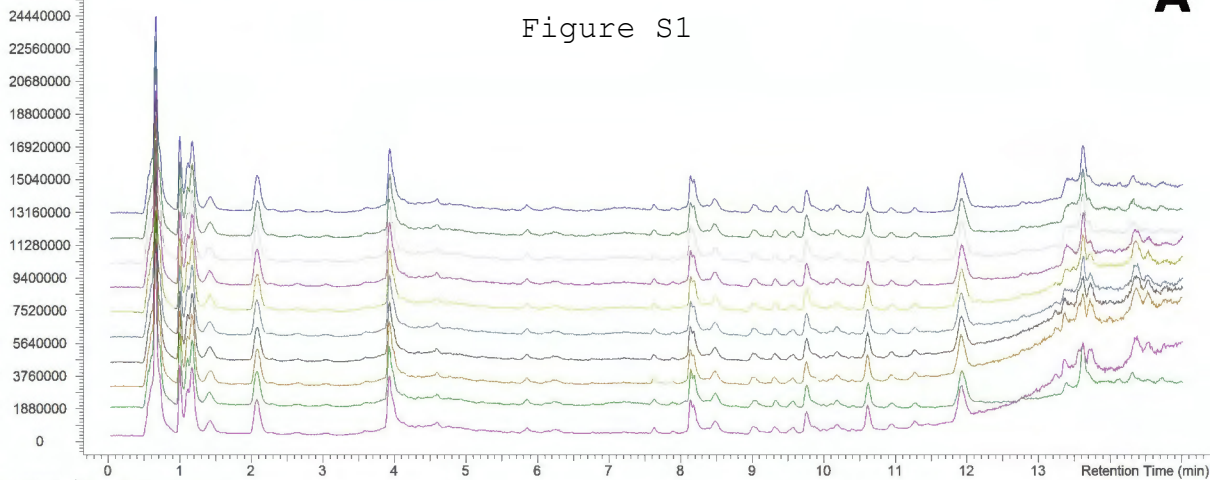

QC-neg-10\_1\_ESI-\_TIC

**B**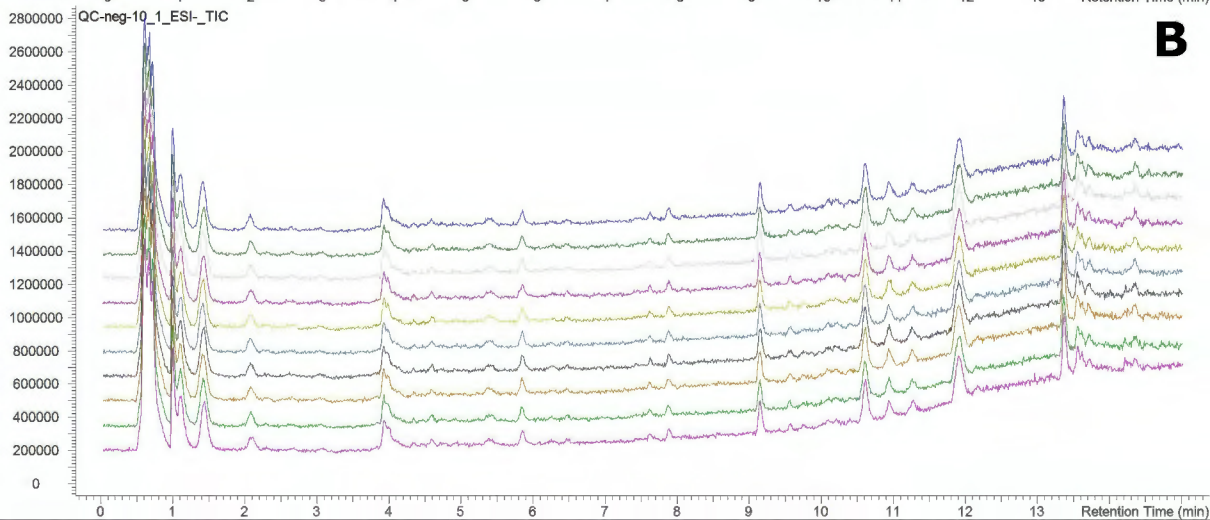

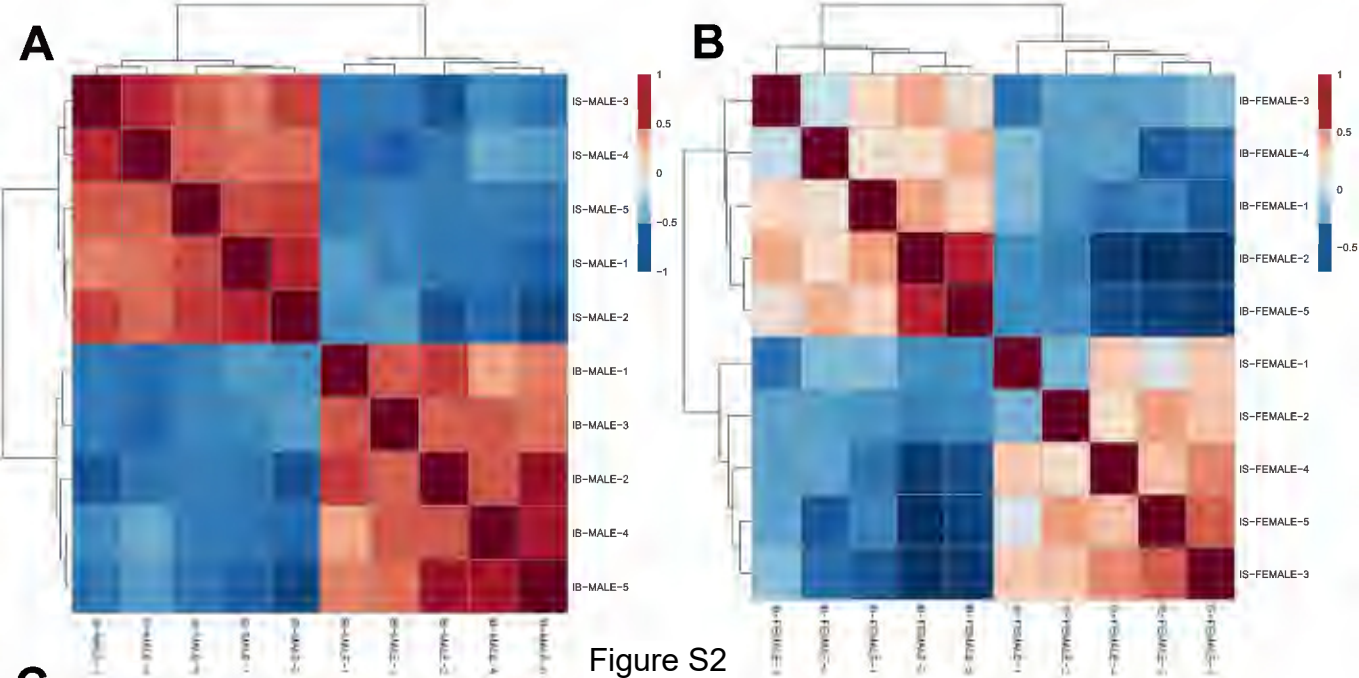

Figure S2

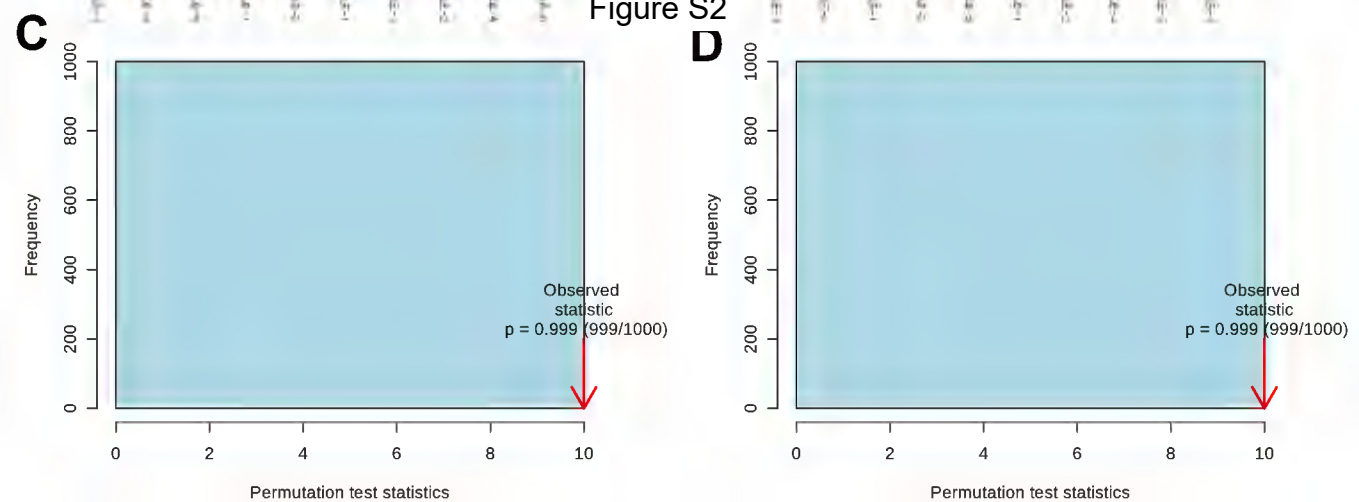

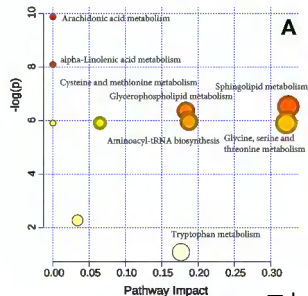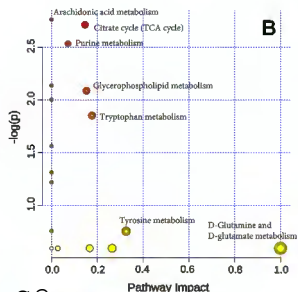

Figure S3

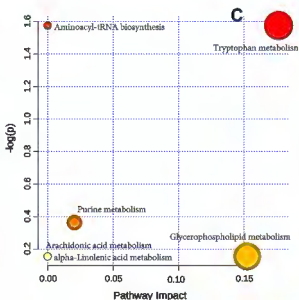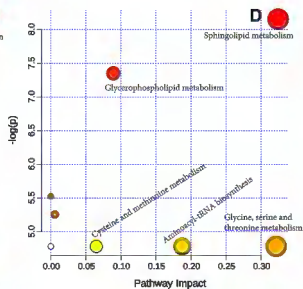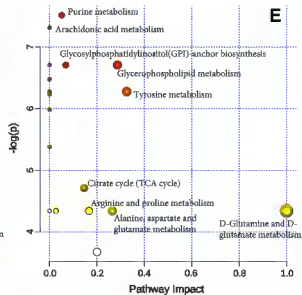

Supplement: FIGURE S1 — The stacked total ion chromatograms of QC sample. (A) The stacked total ion chromatogram of QC sample in ESI+ mode. (B) The stacked total ion chromatogram of QC sample in ESI- mode. The bars on x-axis represent the retention time (0∼15 min) and the bars on y-axis represent the total ion strength. The sample size is 5 in each group of worm samples. [file Data_Sheet_1.PDF]
