# Supplementary material for: Comparative Metabonomic Investigations of Schistosoma japonicum From SCID Mice and BALB/c Mice: Clues to Developmental Abnormality of Schistosome in the Immunodeficient Host
Source: Front Microbiol. 2019 Mar 12;10:440. doi: 10.3389/fmicb.2019.00440 (PMC6423161; doi:10.3389/fmicb.2019.00440)
Supplement: TABLE S1 — Differential metabolites of male worms from SCID mice compared with those from BALB/c mice. This table contains a list of the differential metabolites between male worms from SCID mice and male worms from BALB/c mice. [file Data_Sheet_2.pdf]

Table S1 Differential metabolites of male worms from SCID mice compared with those from BALB/c mice

ESI Mode	m/z	RT(min)	VIP	*P* values	FDR	FC (M_{IS}/M_{IB})	Metabolites	HMDB ID	Chemical formula	Mass (Da)	Adduct	Class	Related pathway
+	860.6126	14.5507	2.32	0.00236	0.005349	17.53	PC(22:6/20:1)[b]	HMDB08735	$C_{50}H_{86}NO_8P$	859.6091	[M+H]	Glycerophospholipids	Glycerophospholipid metabolism
-	118.0509	0.6543	1.22	0.017517	0.023823	1.88	L-Allothreonine[b]	HMDB04041	$C_4H_9NO_3$	119.0582	[M-H]-	Carboxylic acids and derivatives	Glycine, serine and threonine metabolism
-	104.0354	0.6497	1.17	0.008371	0.013552	1.81	L-Serine[a]	HMDB00187	$C_3H_7NO_3$	105.0426	[M-H]-	Carboxylic acids and derivatives	Glycine, serine and threonine metabolism
+	258.1101	0.6723	1.12	0.004564	0.008621	1.78	Glycerophosphocholine[b]	HMDB00086	$C_8H_{20}NO_6P$	257.1028	[M+H]+	Glycerophospholipids	Glycerophospholipid metabolism
+	296.0658	0.6469	0.99	0.00484	0.008661	1.57	5-Aminoimidazole ribonucleotide[b]	HMDB01235	$C_8H_{14}N_3O_7P$	295.0569	[M+H]+	Organooxygen compounds	Purine metabolism
-	179.0556	0.654	0.86	0.049459	0.049459	1.27	L-(+)-Gulose[b]	HMDB12326	$C_6H_{12}O_6$	180.0634	[M-H]-	Organooxygen compounds	Ascorbate and aldarate metabolism
-	102.0549	0.6795	0.85	0.01748	0.023823	1.26	N-Ethylglycine[b]	HMDB41945	$C_4H_9NO_2$	103.0633	[M-H]-	Carboxylic acids and derivatives	Thiamine metabolism
-	124.0058	0.6871	0.73	0.003978	0.007956	1.26	Taurine[a]	HMDB00251	$C_2H_7NO_3S$	125.0147	[M-H]-	Organic sulfonic acids and derivatives	Taurine and hypotaurine metabolism
-	199.0373	0.6377	0.65	0.041292	0.043873	1.21	L-Fucose[b]	HMDB00174	$C_6H_{12}O_5$	164.0685	M+Cl	Organooxygen compounds	Fructose and mannose metabolism
+	146.1176	3.6118	0.80	0.04623	0.047631	0.79	Acetylcholine[b]	HMDB00895	$C_7H_{15}NO_2$	145.1103	[M+H]+	Organonitrogen	Glycerophosph

											compounds	olipid metabolism
+	152.0568	1.4347	0.69	0.022438	0.027246	0.79	Guanine[a]	HMDB00132	$C_5H_5N_5O$	151.0494	[M+H]+	Imidazopyrimidines	Purine metabolism
+	282.2795	9.3158	0.76	0.003731	0.007928	0.78	Oleamide[a]	HMDB02117	$C_{18}H_{35}NO$	281.2719	[M+H]+	Fatty Acyls	——
+	300.2902	9.3158	0.73	0.000153	0.001038	0.78	Sphingosine[a]	HMDB00252	$C_{18}H_{37}NO_2$	299.2824	[M+H]+	Organonitrogen compounds	Sphingolipid metabolism
+	205.0975	3.9998	0.78	0.009361	0.013994	0.75	L-Tryptophan[a]	HMDB00929	$C_{11}H_{12}N_2O_2$	204.0899	[M+H]+	Indoles and derivatives	Tryptophan metabolism
+	482.3605	10.8804	0.89	0.009467	0.013994	0.73	Lyso-PAF C-16[a]	HMDB62689	$C_{24}H_{52}NO_6P$	481.3532	[M+H]+	Glycerophospholipids	Phospholipid metabolism
+	508.3405	12.2338	0.83	0.001437	0.004072	0.73	Gymnodimine[b]	HMDB41430	$C_{25}H_{50}NO_7P$	507.3349	[M+H]+	Not classified	Phospholipid metabolism
+	576.4024	12.5121	0.87	0.000271	0.001152	0.72	LysoPC(22:2)[b]	HMDB10400	$C_{30}H_{58}NO_7P$	575.3951	[M+H]+	Glycerophospholipids	Glycerophospholipid metabolism
+	520.3399	10.1254	0.86	0.001677	0.004386	0.72	PC(18:2/0:0)[a]	HMDB10386	$C_{26}H_{50}NO_7P$	519.3325	[M+H]+	Glycerophospholipids	Phospholipid metabolism
+	454.2931	10.5736	1.06	0.000823	0.002798	0.72	PE(16:0/0:0)[a]	HMDB11503	$C_{21}H_{44}NO_7P$	453.2855	[M+H]+	Glycerophospholipids	Phospholipid metabolism
+	302.3053	9.016	1.23	0.000153	0.001038	0.67	Sphinganine[a]	HMDB00269	$C_{18}H_{39}NO_2$	301.2981	[M+H]+	Organonitrogen compounds	Sphingolipid metabolism
+	552.4029	13.4095	0.94	0.001334	0.004072	0.67	PAF C-18[a]	HMDB62689	$C_{28}H_{58}NO_7P$	551.3951	[M+H]+	Glycerophospholipids	Phospholipid metabolism
+	578.4181	13.7448	1.01	7.20E-05	0.000612	0.66	LysoPC(22:1)[b]/ PC(22:1/0:0)	HMDB10399	$C_{30}H_{60}NO_7P$	577.4107	[M+H]+	Glycerophospholipids	Glycerophospholipid metabolism
+	548.3715	11.2785	1.00	0.000244	0.001152	0.65	LysoPC(20:2)[b]/ PC(O-18:2/2:0)/	HMDB10400	$C_{28}H_{54}NO_7P$	547.3638	[M+H]+	Glycerophospholipids	Glycerophospholipid

							PC(20:2/0:0)						metabolism
+	460.2796	10.9475	1.11	1.99E-05	0.000546	0.63	PE(P-16:0/0:0)[b]	HMDB11152	$C_{21}H_{44}NO_6P$	437.2906	[M+Na]+	Glycerophospholipids	Phospholipid metabolism
+	496.3402	10.5067	1.06	0.000265	0.001152	0.62	PC(16:0/0:0)[a]	HMDB07994	$C_{24}H_{50}NO_7P$	495.3325	[M+H]+	Glycerophospholipids	Phospholipid metabolism
+	524.3717	11.9213	1.05	0.000386	0.001458	0.62	PAF C-16[a]	HMDB62195	$C_{26}H_{54}NO_7P$	523.3638	[M+H]+	Glycerophospholipids	Phospholipid metabolism
+	480.309	10.8995	1.13	4.90E-05	0.000556	0.61	PE(18:1/0:0)[a]	HMDB11506	$C_{23}H_{46}NO_7P$	479.3012	[M+H]+	Glycerophospholipids	Phospholipid metabolism
+	268.1045	1.2762	1.08	0.005214	0.008864	0.57	Adenosine[a]	HMDB00050	$C_{10}H_{13}N_5O_4$	267.0968	[M+H]+	Purine nucleosides	Purine metabolism
+	550.387	12.1807	1.25	3.21E-05	0.000546	0.54	Butenoyl PAF[a]	NA	$C_{28}H_{56}NO_7P$	549.3794	[M+H]+	Glycerophospholipids	Phospholipid metabolism

ESI mode: +, positive ion mode; -, negative ion mode. m/z: mass-to-charge ratio. RT: retention time. FDR: false discovery rate. HMDB: the human metabolome database. FC: fold change. NA: not available. The superscript '[a]': identified by both precise molecular weight and MS/MS spectral alignment. The superscript '[b]': identified by precise molecular weight alignment.

Table S2 Differential metabolites of female worms from SCID mice compared with those from BALB/c mice

ESI Mode	m/z	RT(min)	VIP	*P* values	FDR	FC (F_{IS}/F_{IB})	Metabolites	HMDB ID	Chemical formula	Mass (Da)	Adduct	Class	Related pathway
-	301.2171	12.9477	1.76	0.000132	0.001233	2.34	Retinyl ester[b]	HMDB03598	$C_{20}H_{30}O_2$	302.2246	[M-H]-	Prenol lipids	Retinol metabolism/ Vitamin digestion and absorption
+	269.0885	1.4201	0.72	0.003199	0.006398	0.80	Arabinosylhypoxanthine[a]	HMDB03040	$C_{10}H_{12}N_4O_5$	268.0808	[M+H]+	Purine nucleosides	——
-	464.3143	12.3082	0.96	0.025349	0.027741	0.80	PC(P-15:0/0:0)[b]/PE(P-18:0/ 0:0)[b]/PE(O-18:1/0:0)[b]	NA	$C_{23}H_{48}NO_6P$	465.3219	[M-H]-	Glycerophospholipids	Phospholipid metabolism
+	137.046	1.4199	0.76	0.000225	0.001573	0.79	Hypoxanthine[a]	HMDB00157	$C_5H_4N_4O$	136.0385	[M+H]+	Imidazopyrimidines	Purine metabolism
-	191.0178	0.8004	0.70	0.008998	0.011998	0.79	Citric acid[b]/ Isocitric acid[b]/ Diketogulonic acid[b]/ 2,3-Diketo-L-gulonate[b]	HMDB00094 HMDB00193 HMDB05971 HMDB06511	$C_6H_8O_7$	192.0270	[M-H]-	Carboxylic acids and derivatives	Citrate cycle (TCA cycle)
-	596.3926	13.4124	0.73	0.010324	0.013139	0.79	CerP(18:1/12:0)[b]	HMDB10699	$C_{30}H_{60}NO_6P$	561.4158	[M+Cl]+	Sphingolipids	——
+	284.0993	1.4349	0.77	0.025797	0.027741	0.78	Guanosine[a]	HMDB00133	$C_{10}H_{13}N_5O_5$	283.0917	[M+H]+	Purine nucleosides	Purine metabolism
+	298.0974	4.0607	0.75	0.00461	0.007822	0.78	5'-Methylthioadenosine[a]	HMDB01173	$C_{11}H_{15}N_5O_3S$	297.0896	[M+H]+	5'-deoxyribonucleosid es	Cysteine and methionine metabolism
+	123.0554	1.0077	0.82	0.001965	0.004584	0.77	Niacinamide[a]	HMDB01406	$C_6H_6N_2O$	122.0480	[M+H]+	Pyridines and derivatives	Nicotinate and nicotinamide metabolism
-	267.0717	1.4171	0.80	0.002114	0.004735	0.76	Inosine[a]	HMDB00195	$C_{10}H_{12}N_4O_5$	268.0808	[M-H]-	Purine nucleosides	Purine metabolism
-	256.0576	0.6944	0.79	0.007146	0.010815	0.76	N-Acetylmannosamine[b]/N-A cetyl-b-D-galactosamine[b]/B eta-N-Acetylglucosamine[b]/N -Acetylgalactosamine[b]	HMDB01129 HMDB00853 HMDB00803 HMDB00212	$C_8H_{15}NO_6$	221.0899	[M+Cl]+	Organooxygen compounds	Amino sugar and nucleotide sugar metabolism
+	152.0568	1.4347	0.82	0.003534	0.006597	0.74	Guanine[a]	HMDB00132	$C_5H_5N_5O$	151.0494	[M+H]+	Imidazopyrimidines	Purine metabolism
+	552.4029	13.4095	0.83	0.012797	0.01586	0.72	PAF C-18[a]	HMDB62689	$C_{28}H_{58}NO_7P$	551.3951	[M+H]+	Glycerophospholipids	Phospholipid

												metabolism
-	219.0968	0.6785	0.86	0.0246	0.027552	0.72	N-Acetyl-b-glucosaminylamine[b]	HMDB00853	$C_8H_{16}N_2O_5$	220.1059	[M-H]-	Organooxygen compounds	——
-	327.2327	13.3711	0.90	0.00792	0.011671	0.71	Docosahexaenoic acid[b]	HMDB02183	$C_{22}H_{32}O_2$	328.2402	[M-H]-	Fatty Acyls	Biosynthesis of unsaturated fatty acids
-	592.3605	11.2786	0.90	0.004009	0.007241	0.69	PC(16:0/5:0(CHO))[b]	NA	$C_{29}H_{56}NO_9P$	593.3693	[M-H]-	Glycerophospholipids	Phospholipid metabolism
+	132.1019	1.172	0.91	0.004177	0.00731	0.68	L-Isoleucine[a]	HMDB00172	$C_6H_{13}NO_2$	131.0946	[M+H]+	Carboxylic acids and derivatives	Valine, leucine and isoleucine degradation
-	480.309	11.876	0.96	0.00122	0.003416	0.67	LysoPC(15:0)[b]/LysoPE(0:0/18:0)[b]/PC(14:0/O-1:0)[b]/PC(7:0/O-8:0)[b]/PE(18:0/0:0)[b]/PC(15:0/0:0)[b]	HMDB10381 HMDB11129	$C_{23}H_{48}NO_7P$	481.3168	[M-H]-	Glycerophospholipids	Glycerophospholipid metabolism
-	293.1175	4.4634	0.95	0.003346	0.006462	0.67	Glutamylphenylalanine[b]	HMDB00594	$C_{14}H_{18}N_2O_5$	294.1216	[M-H]-	Carboxylic acids and derivatives	——
-	557.3195	10.6175	1.00	0.000432	0.001862	0.66	Oleic Acid-biotin[b]	HMDB00207 HMDB00030	$C_{28}H_{50}N_4O_3S$	522.3604	[M+Cl]+	Fatty Acyls	——
+	182.0811	1.1304	0.98	0.001879	0.004574	0.65	L-Tyrosine[a]	HMDB00158	$C_9H_{11}NO_3$	181.0739	[M+H]+	Carboxylic acids and derivatives	Tyrosine metabolism
-	436.2818	10.9465	1.01	0.000269	0.001573	0.65	PE(P-16:0e/0:0)[b]	HMDB11152	$C_{21}H_{44}NO_6P$	437.2906	[M-H]-	Glycerophospholipids	Phospholipid metabolism
-	146.0449	0.6795	1.00	0.013028	0.01586	0.64	L-Glutamic acid[a]	HMDB00148	$C_5H_9NO_4$	147.0532	[M-H]-	Carboxylic acids and derivatives	Glutathione metabolism
+	548.3715	11.2785	1.03	0.001202	0.003416	0.64	LysoPC(20:2)[b]/PC(O-18:2/2:0)[b]/PC(20:2/0:0)[b]	HMDB10392	$C_{28}H_{54}NO_7P$	547.3638	[M+H]+	Glycerophospholipids	Glycerophospholipid metabolism
-	540.3297	10.6169	1.03	0.000736	0.002944	0.64	(25R)-3alpha,7alpha-dihydroxy-5beta-cholestan-27-oyl	HMDB10408	$C_{29}H_{51}NO_6S$	541.3437	[M-H]-	Glycerophospholipids	——

							taurine[b]/LysoPC(P-18:1)[b]/C-8 Ceramide-1-phosphate[b]						
+	524.3717	11.9213	1.05	0.000301	0.001573	0.63	PAF C-16[b]	HMDB62195	$C_{26}H_{54}NO_7P$	523.3638	[M+H]+	Glycerophospholipids	Phospholipid metabolism
-	452.2769	10.5719	1.03	0.002999	0.006219	0.63	LysoPE(0:0/16:0)[b]/PE(16:0/0:0)[b]/PC(13:0/0:0)[b]	HMDB11473	$C_{21}H_{44}NO_7P$	453.2855	[M-H]-	Glycerophospholipids	Phospholipid metabolism
-	281.2481	14.5325	1.08	7.09E-05	0.000794	0.63	Vaccenic acid[b]/Oleic acid[b]	HMDB03231 HMDB00207	$C_{18}H_{34}O_2$	282.2559	[M-H]-	Fatty Acyls	——
+	205.0975	3.9998	1.07	0.00142	0.003787	0.61	L-Tryptophan[a]	HMDB00929	$C_{11}H_{12}N_2O_2$	204.0899	[M+H]+	Indoles and derivatives	Tryptophan metabolism
+	454.2931	10.5736	1.10	0.00111	0.003416	0.60	PE(16:0/0:0)[a]	HMDB11503	$C_{21}H_{44}NO_7P$	453.2855	[M+H]+	Glycerophospholipids	Phospholipid metabolism
-	535.1528	1.4173	1.12	0.001719	0.004375	0.59	1,4-beta-D-Glucan[b]/Isocaviunin 7-O-glucoside[b]	HMDB06944	$C_{18}H_{32}O_{18}$	536.1589	[M-H]-	Organooxygen compounds	Carbohydrate digestion and absorption
+	146.0602	4.0002	1.13	0.000858	0.003004	0.58	4-formyl Indole[a]	HMDB29737	C_9H_7NO	145.0528	[M+H]+	Indoles and derivatives	——
+	550.387	12.1807	1.16	0.000218	0.001573	0.57	Butenoyl PAF[a]	NA	$C_{28}H_{56}NO_7P$	549.3794	[M+H]+	Glycerophospholipids	Phospholipid metabolism
+	578.4181	13.7448	1.19	4.00E-05	0.000746	0.57	LysoPC(22:1)[b]/PC(22:1/0:0)[b]	HMDB10399	$C_{30}H_{60}NO_7P$	577.4107	[M+H]+	Glycerophospholipids	Glycerophospholipid metabolism
-	303.2326	13.5603	1.185	0.000836	0.003004	0.56	Arachidonic acid[b]	HMDB01043	$C_{20}H_{32}O_2$	304.2402	[M-H]-	Fatty Acyls	Arachidonic acid metabolism
-	478.2923	10.9031	0.96	0.000403	0.001862	0.55	LysoPE(0:0/18:1)[a]	HMDB11475	$C_{23}H_{46}NO_7P$	479.3012	[M-H]-	Glycerophospholipids	Phospholipid metabolism
-	331.2635	14.2904	1.27	0.000309	0.001573	0.51	Adrenic acid[b]	HMDB02226	$C_{22}H_{36}O_2$	332.2715	[M-H]-	Fatty Acyls	Arachidonic acid metabolism
-	264.005	0.7038	1.36	2.66E-05	0.000745	0.50	5-Phosphoribosylamine[b]	HMDB01128	$C_5H_{12}NO_7P$	229.0351	[M+Cl]+	Organooxygen compounds	Purine metabolism

+	496.3402	10.5067	1.30	0.024157	0.027552	0.47	PC(16:0/0:0)[a]	HMDB07994	$C_{24}H_{50}NO_7P$	495.3325	[M+H]+	Glycerophospholipids	Phospholipid metabolism
+	860.6126	14.5507	1.98	0.009766	0.012718	0.31	PC(22:6/20:1)[b]	HMDB08735	$C_{50}H_{86}NO_8P$	859.6091	[M+H]	Glycerophospholipids	Glycerophospholipid metabolism
+	230.0967	0.7226	1.83	7.35E-07	4.12E-05	0.29	Ergothioneine[a]	HMDB03045	$C_9H_{15}N_3O_2S$	229.0885	[M+H]+	Carboxylic acids and derivatives	Histidine metabolism

ESI mode: +, positive ion mode; -, negative ion mode. m/z: mass-to-charge ratio. RT: retention time. FDR: false discovery rate. HMDB: the human metabolome database. FC: fold change. NA: not available. The superscript ‘[a]’: identified by both precise molecular weight and MS/MS spectral alignment. The superscript ‘[b]’: identified by precise molecular weight alignment.

Table S3 Metabolite set enrichment of the differential metabolites of male worms between SCID mice and BALB/c mice

Metabolite set	Total Cmpd	Hits	Statistic Q	Expected Q	Raw p	Holm p	FDR
Bile Acid Biosynthesis	65	1	82.067	11.111	0.00030572	0.0045858	0.0022929
Taurine and Hypotaurine Metabolism	12	1	82.067	11.111	0.00030572	0.0045858	0.0022929
Sphingolipid Metabolism	40	3	56.355	11.111	0.0014637	0.019028	0.0038041
Retinol Metabolism	37	1	73.007	11.111	0.0016413	0.019696	0.0038041
Purine Metabolism	74	3	55.826	11.111	0.002247	0.024717	0.0038041
Fructose and Mannose Degradation	32	1	70.837	11.111	0.002262	0.024717	0.0038041
Ammonia Recycling	32	1	69.506	11.111	0.0027233	0.024717	0.0038041
Glycine and Serine Metabolism	59	1	69.506	11.111	0.0027233	0.024717	0.0038041
Homocysteine Degradation	9	1	69.506	11.111	0.0027233	0.024717	0.0038041
Phosphatidylethanolamine Biosynthesis	12	1	69.506	11.111	0.0027233	0.024717	0.0038041
Methionine Metabolism	43	2	56.973	11.111	0.0030432	0.024717	0.0038041
Selenoamino Acid Metabolism	28	2	56.973	11.111	0.0030432	0.024717	0.0038041
Betaine Metabolism	21	1	29.898	11.111	0.10192	0.30577	0.11761
Tryptophan Metabolism	60	1	11.464	11.111	0.33857	0.67715	0.36276
Phospholipid Biosynthesis	29	2	9.66	11.111	0.4001	0.67715	0.4001

FDR: false discovery rate.

Table S4 Metabolite set enrichment of the differential metabolites of female worms between SCID mice and BALB/c mice

Metabolite set	Total Cmpd	Hits	Statistic Q	Expected Q	Raw p	Holm p	FDR
Retinol Metabolism	37	1	95.973	11.111	7.31E-07	2.92E-05	2.92E-05
Alpha Linolenic Acid and Linoleic Acid Metabolism	19	3	51.676	11.111	0.0054066	0.21086	0.10813
Purine Metabolism	74	6	33.158	11.111	0.010014	0.38054	0.11067
Sphingolipid Metabolism	40	1	57.46	11.111	0.011067	0.4095	0.11067
Glutamate Metabolism	49	3	36.266	11.111	0.019396	0.69827	0.15517
Arachidonic Acid Metabolism	69	2	29.616	11.111	0.055047	1	0.33928
Citric Acid Cycle	32	3	31.199	11.111	0.063098	1	0.33928
Transfer of Acetyl Groups into Mitochondria	22	2	28.151	11.111	0.067857	1	0.33928
Phospholipid Biosynthesis	29	1	31.923	11.111	0.088777	1	0.39457
Warburg Effect	58	4	21.99	11.111	0.12858	1	0.51431
Methionine Metabolism	43	1	18.879	11.111	0.20955	1	0.59704
Spermidine and Spermine Biosynthesis	18	1	18.879	11.111	0.20955	1	0.59704
Amino Sugar Metabolism	33	2	16.281	11.111	0.23555	1	0.59704
Biotin Metabolism	8	1	14.998	11.111	0.26887	1	0.59704
Fatty Acid Biosynthesis	35	1	14.998	11.111	0.26887	1	0.59704
Gluconeogenesis	35	1	14.998	11.111	0.26887	1	0.59704
Pyruvate Metabolism	48	1	14.998	11.111	0.26887	1	0.59704
Threonine and 2-Oxobutanoate Degradation	20	1	14.998	11.111	0.26887	1	0.59704
Tryptophan Metabolism	60	2	13.512	11.111	0.31711	1	0.59704
Catecholamine Biosynthesis	20	1	6.716	11.111	0.46967	1	0.59704
Thyroid hormone synthesis	13	1	6.716	11.111	0.46967	1	0.59704
Nicotinate and Nicotinamide Metabolism	37	2	8.7177	11.111	0.50669	1	0.59704
Alanine Metabolism	17	2	7.7593	11.111	0.52973	1	0.59704
Ammonia Recycling	32	2	7.7593	11.111	0.52973	1	0.59704
Propanoate Metabolism	42	2	7.7593	11.111	0.52973	1	0.59704
Arginine and Proline Metabolism	53	1	4.5902	11.111	0.55226	1	0.59704
Aspartate Metabolism	35	1	4.5902	11.111	0.55226	1	0.59704
Beta-Alanine Metabolism	34	1	4.5902	11.111	0.55226	1	0.59704
Cysteine Metabolism	26	1	4.5902	11.111	0.55226	1	0.59704
Folate Metabolism	29	1	4.5902	11.111	0.55226	1	0.59704
Glucose-Alanine Cycle	13	1	4.5902	11.111	0.55226	1	0.59704
Glutathione Metabolism	21	1	4.5902	11.111	0.55226	1	0.59704
Glycine and Serine Metabolism	59	1	4.5902	11.111	0.55226	1	0.59704

Histidine Metabolism	43	1	4.5902	11.111	0.55226	1	0.59704
Lysine Degradation	30	1	4.5902	11.111	0.55226	1	0.59704
Malate-Aspartate Shuttle	10	1	4.5902	11.111	0.55226	1	0.59704
Urea Cycle	29	1	4.5902	11.111	0.55226	1	0.59704
Phenylalanine and Tyrosine Metabolism	28	2	5.4709	11.111	0.63938	1	0.65578
Tyrosine Metabolism	72	2	5.4709	11.111	0.63938	1	0.65578
Valine, Leucine and Isoleucine Degradation	60	3	5.2154	11.111	0.72927	1	0.72927

FDR: false discovery rate.

Table S5 Metabolite set enrichment of the common differential metabolites in male and female worms from SCID mice

Metabolite set	Total Cmpd	Hits	Statistic Q	Expected Q	Raw p	Holm p	FDR
Phospholipid Biosynthesis	29	1	42.655	11.111	4.06E-02	1.22E-01	1.22E-01
Tryptophan Metabolism	60	1	19.068	11.111	0.20703	0.41407	0.31055
Purine Metabolism	74	1	2.0129	11.111	0.69582	0.69582	0.69582

FDR: false discovery rate.

Table S6 Metabolite set enrichment of the male worm-specific differential metabolites between SCID mice and BALB/c mice

Metabolite set	Total Cmpd	Hits	Statistic Q	Expected Q	Raw p	Holm p	FDR
Sphingolipid Metabolism	40	3	69.59	11.111	0.00028738	0.0040233	0.0040233
Purine Metabolism	74	2	64.67	11.111	0.0029258	0.038036	0.009125
Methionine Metabolism	43	2	62.157	11.111	0.0034006	0.040808	0.009125
Selenoamino Acid Metabolism	28	2	62.157	11.111	0.0034006	0.040808	0.009125
Bile Acid Biosynthesis	65	1	66.607	11.111	0.0039794	0.040808	0.009125
Taurine and Hypotaurine Metabolism	12	1	66.607	11.111	0.0039794	0.040808	0.009125
Retinol Metabolism	37	1	65.493	11.111	0.0045663	0.040808	0.009125
Betaine Metabolism	21	1	64.386	11.111	0.0052143	0.040808	0.009125
Ammonia Recycling	32	1	60.165	11.111	0.0083685	0.050211	0.0097633
Glycine and Serine Metabolism	59	1	60.165	11.111	0.0083685	0.050211	0.0097633
Homocysteine Degradation	9	1	60.165	11.111	0.0083685	0.050211	0.0097633
Phosphatidylethanolamine Biosynthesis	12	1	60.165	11.111	0.0083685	0.050211	0.0097633
Fructose and Mannose Degradation	32	1	42.436	11.111	0.041298	0.082596	0.044475
Phospholipid Biosynthesis	29	1	40.979	11.111	0.046188	0.082596	0.046188

FDR: false discovery rate.

Table S7 Metabolite set enrichment of the female worm-specific differential metabolites of those between SCID mice and BALB/c mice

Metabolite set	Total Cmpd	Hits	Statistic Q	Expected Q	Raw p	Holm p	FDR
Retinol Metabolism	37	1	85.405	11.111	1.32E-04	4.62E-03	3.19E-03
Purine Metabolism	74	5	74.273	11.111	0.00018248	0.0062042	0.0031933
Glutamate Metabolism	49	2	75.045	11.111	0.00054561	0.018005	0.0063654
Alpha Linolenic Acid and Linoleic Acid Metabolism	19	3	75.161	11.111	0.00080408	0.025731	0.0070357
Warburg Effect	58	3	57.679	11.111	0.0015392	0.047717	0.0078907
Catecholamine Biosynthesis	20	1	72.11	11.111	0.0018794	0.056381	0.0078907
Thyroid hormone synthesis	13	1	72.11	11.111	0.0018794	0.056381	0.0078907
Arachidonic Acid Metabolism	69	2	67.246	11.111	0.0018812	0.056381	0.0078907
Nicotinate and Nicotinamide Metabolism	37	2	61.485	11.111	0.002029	0.056381	0.0078907
Phospholipid Biosynthesis	29	1	68.793	11.111	0.002999	0.077974	0.0093771
Valine, Leucine and Isoleucine Degradation	60	2	60.47	11.111	0.0034709	0.086772	0.0093771
Phenylalanine and Tyrosine Metabolism	28	2	63.346	11.111	0.0034829	0.086772	0.0093771
Tyrosine Metabolism	72	2	63.346	11.111	0.0034829	0.086772	0.0093771
Amino Sugar Metabolism	33	2	57.979	11.111	0.0038513	0.086772	0.0096282
Methionine Metabolism	43	1	65.417	11.111	0.0046088	0.096784	0.010082
Spermidine and Spermine Biosynthesis	18	1	65.417	11.111	0.0046088	0.096784	0.010082
Citric Acid Cycle	32	2	59.477	11.111	0.0089993	0.17099	0.013031
Transfer of Acetyl Groups into Mitochondria	22	1	59.477	11.111	0.0089993	0.17099	0.013031
Sphingolipid Metabolism	40	1	58.15	11.111	0.010322	0.17548	0.013031
Alanine Metabolism	17	1	55.805	11.111	0.013031	0.2085	0.013031
Ammonia Recycling	32	1	55.805	11.111	0.013031	0.2085	0.013031
Arginine and Proline Metabolism	53	1	55.805	11.111	0.013031	0.2085	0.013031
Aspartate Metabolism	35	1	55.805	11.111	0.013031	0.2085	0.013031
Beta-Alanine Metabolism	34	1	55.805	11.111	0.013031	0.2085	0.013031
Cysteine Metabolism	26	1	55.805	11.111	0.013031	0.2085	0.013031
Folate Metabolism	29	1	55.805	11.111	0.013031	0.2085	0.013031
Glucose-Alanine Cycle	13	1	55.805	11.111	0.013031	0.2085	0.013031
Glutathione Metabolism	21	1	55.805	11.111	0.013031	0.2085	0.013031
Glycine and Serine Metabolism	59	1	55.805	11.111	0.013031	0.2085	0.013031
Histidine Metabolism	43	1	55.805	11.111	0.013031	0.2085	0.013031
Lysine Degradation	30	1	55.805	11.111	0.013031	0.2085	0.013031
Malate-Aspartate Shuttle	10	1	55.805	11.111	0.013031	0.2085	0.013031

Propanoate Metabolism	42	1	55.805	11.111	0.013031	0.2085	0.013031
Tryptophan Metabolism	60	1	55.805	11.111	0.013031	0.2085	0.013031
Urea Cycle	29	1	55.805	11.111	0.013031	0.2085	0.013031

FDR: false discovery rate.

Table S8 Pathway analysis of the differential metabolites of male worms between SCID mice and BALB/c mice

Metabolic pathways	Hits/Total	Raw p	Holm adjust	FDR	Impact
Arachidonic acid metabolism	1/14	5.12E-05	0.00061387	0.00030693	0
alpha-Linolenic acid metabolism	1/3	5.12E-05	0.00061387	0.00030693	0
Taurine and hypotaurine metabolism	1/6	0.00030572	0.0030572	0.0012229	0
Sphingolipid metabolism	3/17	0.0014637	0.013173	0.003268	0.32433
Glycerophospholipid metabolism	4/31	0.0017247	0.013798	0.003268	0.18323
Aminoacyl-tRNA biosynthesis	2/46	0.0025849	0.018094	0.003268	0.1875
Glycine, serine and threonine metabolism	1/19	0.0027233	0.018094	0.003268	0.32151
Cysteine and methionine metabolism	1/21	0.0027233	0.018094	0.003268	0.06486
Cyanoamino acid metabolism	1/6	0.0027233	0.018094	0.003268	0
Glyoxylate and dicarboxylate metabolism	1/17	0.0027233	0.018094	0.003268	0
Purine metabolism	2/49	0.10318	0.20637	0.11256	0.03394
Tryptophan metabolism	1/24	0.33857	0.33857	0.33857	0.17647

FDR: false discovery rate.

Table S9 Pathway analysis of the differential metabolites of female worms between SCID mice and BALB/c mice

Metabolic pathways	Hits/Total	Raw p	Holm adjust	FDR	Impact
Biotin metabolism	1/8	0.0004322	0.0082118	0.0046809	0
Tryptophan metabolism	1/24	0.00142	0.025561	0.0046809	0.17647
Purine metabolism	4/49	0.0014582	0.025561	0.0046809	0.07208
Glyoxylate and dicarboxylate metabolism	3/17	0.0015391	0.025561	0.0046809	0
Tyrosine metabolism	1/15	0.0018786	0.028179	0.0046809	0.32558
Phenylalanine, tyrosine and tryptophan biosynthesis	1/4	0.0018786	0.028179	0.0046809	0
Nicotinate and nicotinamide metabolism	1/9	0.0019646	0.028179	0.0046809	0
Aminoacyl-tRNA biosynthesis	4/46	0.0019709	0.028179	0.0046809	0
Cysteine and methionine metabolism	1/21	0.0046096	0.050705	0.0097313	0
Arachidonic acid metabolism	2/14	0.00596	0.0596	0.011324	0
Glycerophospholipid metabolism	2/31	0.0067799	0.061019	0.011711	0.15253
Citrate cycle (TCA cycle)	2/20	0.0089983	0.071987	0.013751	0.14595
D-Glutamine and D-glutamate metabolism	1/5	0.013028	0.091193	0.013751	1
Alanine, aspartate and glutamate metabolism	1/17	0.013028	0.091193	0.013751	0.26471
Arginine and proline metabolism	1/23	0.013028	0.091193	0.013751	0.16667
Glutathione metabolism	1/19	0.013028	0.091193	0.013751	0.02721
Porphyrin and chlorophyll metabolism	1/17	0.013028	0.091193	0.013751	0
Nitrogen metabolism	1/5	0.013028	0.091193	0.013751	0
alpha-Linolenic acid metabolism	1/3	0.024157	0.091193	0.024157	0

ESI mode: +, positive ion mode; -, negative ion mode. m/z: mass-to-charge ratio. RT: retention time. FDR: false discovery rate. FC: fold change. NA: not available. The superscript 'a': identified by both precise molecular weight and MS/MS spectral alignment. The superscript 'b': identified by precise molecular weight alignment.

Table S10 Pathway analysis of the common differential metabolites in male and female worms from SCID mice

Metabolic pathways	Hits/Total	Raw p	Holm adjust	FDR	Impact
Tryptophan metabolism	1/24	0.20703	1	0.6211	0.17647
Aminoacyl-tRNA biosynthesis	1/46	0.20703	1	0.6211	0
Purine metabolism	1/49	0.69582	1	0.85651	0.02052
Glycerophospholipid metabolism	2/31	0.85651	1	0.85651	0.15253
Arachidonic acid metabolism	1/14	0.85651	1	0.85651	0
alpha-Linolenic acid metabolism	1/3	0.85651	1	0.85651	0

FDR: false discovery rate.

Table S11 Pathway analysis of the male worm-specific differential metabolites between SCID mice and BALB/c mice

Metabolic pathways	Hits/Total	Raw p	Holm adjust	FDR	Impact
Sphingolipid metabolism	2/17	0.000227	0.002047	0.002047	0.18919
Glycerophospholipid metabolism	3/31	0.000642	0.005136	0.002889	0.08918
Taurine and hypotaurine metabolism	1/6	0.003979	0.027856	0.008787	0
Purine metabolism	1/49	0.005214	0.031286	0.008787	0.00579
Glycine, serine and threonine metabolism	1/19	0.008369	0.041843	0.008787	0.32151
Cysteine and methionine metabolism	1/21	0.008369	0.041843	0.008787	0.06486
Cyanoamino acid metabolism	1/6	0.008369	0.041843	0.008787	0
Glyoxylate and dicarboxylate metabolism	1/17	0.008369	0.041843	0.008787	0
Aminoacyl-tRNA biosynthesis	2/46	0.008787	0.041843	0.008787	0.1875

FDR: false discovery rate.

Table S12 Pathway analysis of the female worm-specific differential metabolites between SCID mice and BALB/c mice

Metabolic pathways	Hits/Total	Raw p	Holm adjust	FDR	Impact
Arachidonic acid metabolism	2/14	0.000666	0.013317	0.004366	0
Glycerophospholipid metabolism	3/31	0.000899	0.017081	0.004366	0.28655
Glycosylphosphatidylinositol(GPI)-anchor biosynthesis	1/12	0.00122	0.021965	0.004366	0.06897
alpha-Linolenic acid metabolism	1/3	0.00122	0.021965	0.004366	0
Glyoxylate and dicarboxylate metabolism	3/17	0.001539	0.024628	0.004366	0
Tyrosine metabolism	1/15	0.001879	0.02819	0.004366	0.32558
Phenylalanine, tyrosine and tryptophan biosynthesis	1/4	0.001879	0.02819	0.004366	0
Purine metabolism	1/49	0.001902	0.02819	0.004366	0.00415
Nicotinate and nicotinamide metabolism	1/9	0.001965	0.02819	0.004366	0
Aminoacyl-tRNA biosynthesis	3/46	0.002515	0.02819	0.00503	0
Cysteine and methionine metabolism	1/21	0.004609	0.046088	0.00838	0
Citrate cycle (TCA cycle)	2/20	0.008999	0.080994	0.014479	0.14595
D-Glutamine and D-glutamate metabolism	1/5	0.013031	0.10425	0.014479	1
Alanine, aspartate and glutamate metabolism	1/17	0.013031	0.10425	0.014479	0.26471
Arginine and proline metabolism	1/23	0.013031	0.10425	0.014479	0.16667
Glutathione metabolism	1/19	0.013031	0.10425	0.014479	0.02721
Porphyrin and chlorophyll metabolism	1/17	0.013031	0.10425	0.014479	0
Nitrogen metabolism	1/5	0.013031	0.10425	0.014479	0
Ether lipid metabolism	1/10	0.02535	0.10425	0.026684	0.2
Pyrimidine metabolism	1/32	0.0381	0.10425	0.0381	0

FDR: false discovery rate.

Table S13. Information of qPCR primers of the tested genes involved in retinol metabolism and meiosis

Accession number*	Genes**	Primer name	Primer sequence (5’→3’)***	Primer length (nt)	Tm (℃)	Amplicon length (bp)
FN320595	*PSMD4*	SjPSMD4-F	CCTCACCAACAATTTCCACATCT	23	60	129
		SjPSMD4-R	GATCACTTATAGCCTTGCGAACAT	24	60	
Sjp_0052550	*SPO11*	SjSPO11-F	ATTACGGCTAAAGGTTATCCAGA	23	60	104
		SjSPO11-R	GGATCCGCATCAAAAAGTCCA	21	60	
Sjp_0069970/ AY915683.1	*MRE11*	SjMRE11-F	TCGAATGCGTAAAGCTCCAGA	21	60	191
		SjMRE11-R	TTTCCTGTGGTTCTGCTGGT	20	60	
AY223066.1\ AY811553.1	*MND1*	SjMND1-F	CTTACCGCGGAACTTCAGGA	20	60	103
		SjMND1-R	CCGTCCAACGATTAGCAGAGT	21	60	
Sjp_0047790/ AY223000.2	*skp1a*	Sjskp1a-F	CCAGTACCCTTGCCGAATGT	20	60	193
		Sjskp1a-R	TGGCCGCTAACATCAACTCA	20	60	
Sjp_0108620	*DGAT1*	SjDGAT1-F	AGCTGGCTTGGTTGTCTTCA	20	60	161
		SjDGAT1-R	TGCCACCTTGACCACCATTT	20	60	
Sjp_0036830	*RDH12*	SjRDH12-F	TTGCTAGACGAATCCTCGCC	20	60	185
		SjRDH12-R	TCGACATGGAGTTGAGCGTT	20	60	
Sjp_0004470	*DHRS*	SjDHRS-F	GCTGGCTATTGTGCAAGTCG	20	60	164

		SjDHRS-R	TGATCCACGTATGCACCAGT	20	60	
Sjp_0046010/ FN317793.1	*ALDH1B1*	SjALDH1B1-F	ATGAAAGCAGCGGCTAGTAGT	21	60	151
		SjALDH1B1-R	CGGCTACACAACACTGTCCA	20	60	
Sjp_0054160	*PLK4*	SjPLK4-F	CGAGGAGGTTTCGCACAAGT	20	60	119
		SjPLK4-R	CGACGTACACGGTTCGTTAGT	21	60	
FN317236.1	*PLK1*	SjPLK1-F	CCCTCTGATGCATGCCGTAA	20	60	174
		SjPLK1-R	GCTGAGGAAGTTGTTCCCGA	20	60	
Sjp_0042290	*DMC1*	SjDMC1-F	GGCTTACACGGGCTGGATTT	20	60	109
		SjDMC1-R	TCCAGAGCATTACGAGCGAC	20	60	
Sjp_0000050/ AY812723.1	*RAD51*	SjRAD51-F	GAAAGTTGCGGTCCATTGCT	20	60	152
		SjRAD51-R	TCGCTGATTCCTTTTACCGCA	21	60	

*__Accession number__: Accession number of tested genes assigned in ***NCBI*** database (http://www.ncbi.nlm.nih.gov) or ***WormBase ParaSite*** database (https://parasite.wormbase.org/index.html) could be used to retrieve the full or partial sequences of target genes.

**__Genes__: Brief annotation of genes with abbreviated names are described as follow.

PSMD4: 26S proteasome non-ATPase regulatory subunit 4, one of the non-ATPase subunits of the 19S regulator base, subunit Rpn10.

SPO11: a meiosis-specific protein involved in the creation of double stranded breaks in the DNA in the early stages of this process.

MRE11: meiotic recombination repair protein 11, a nuclear protein involved in homologous recombination, telomere length maintenance, and DNA double-strand break repair.

MND1: meiotic nuclear division protein 1-like protein.

skp1a: S-phase kinase-associated protein 1A.

DGAT1: diacylglycerol O-acyltransferase 1. This gene encodes an multipass transmembrane protein that functions as a key metabolic enzyme. The encoded protein catalyzes the conversion of diacylglycerol and fatty acyl CoA to triacylglycerol. This enzyme can also

transfer acyl CoA to retinol.

RDH12: retinol dehydrogenase 12. RDH12 belongs to a family of dual-specificity retinol dehydrogenases that metabolize both all-trans- and cis-retinols.0

DHRS: Retinol dehydrogenase/reductase. This enzyme participates in retinol metabolism.

ALDH1B1: aldehyde dehydrogenase 1B1 precursor/retinol dehydrogenase. This enzyme belongs to the family of oxidoreductases, specifically those acting on the CH-OH group of donor with NAD+ or NADP+ as acceptor. This enzyme participates in retinol metabolism.

PLK4: serine/threonine-protein kinase PLK4, also known as polo-like kinase 4. The *Drosophila* homolog is SAK, the *C. elegans* homolog is zyg-1. The protein localizes to centrioles-complex microtubule-based structures found in centrosomes-and regulates centriole duplication during the cell cycle.

PLK1: serine/threonine-protein kinase PLK1, also known as polo-like kinase 1 (PLK-1) or serine/threonine-protein kinase 13 (STPK13). Plk1 is an early trigger for G2/M transition. Plk1 supports the functional maturation of the centrosome in late G2/early prophase and establishment of the bipolar spindle with a possible role for chromosome separation.

DMC1: "Dmc" stands for "disrupted meiotic cDNA" and refers to the method used for its discovery which involved using clones from a meiosis-specific cDNA library to direct knock-out mutations of abundantly expressed meiotic genes. Meiotic recombination protein Dmc1 plays the central role in homologous recombination in meiosis by assembling at the sites of programmed DNA double strand breaks and carrying out a search for allelic DNA sequences located on homologous chromatids.

RAD51: a eukaryotic gene, encodes a member of the RAD51 protein family which plays a major role in homologous recombination of DNA during double strand break repair.

*** **Primer sequences**: All specific qPCR primers sequences of the tested genes (except for the internal control gene *PSMD4*) were designed by Liu R. using the NCBI/Primer-BLAST (https://www.ncbi.nlm.nih.gov/tools/primer-blast/index.cgi?LINK_LOC=BlastHome) with specific parameters set as PCR amplicon length of 100 - 200 bp, melting temperature (Tm) of approximately 60 °C and primer pair specificity checking against Refseq mRNA (Database) of *Schistosoma* (taxid:6181) (Organism).
